# Preclinical evaluation of the efficacy of an antibody to human SIRPα for cancer immunotherapy in humanized mouse models

**DOI:** 10.3389/fimmu.2023.1294814

**Published:** 2023-12-14

**Authors:** Yasuyuki Saito, Rie Iida-Norita, Tania Afroj, Alaa Refaat, Daisuke Hazama, Satomi Komori, Shinya Ohata, Tomoko Takai, Okechi S. Oduori, Takenori Kotani, Yohei Funakoshi, Yu-Ichiro Koma, Yoji Murata, Kimikazu Yakushijin, Hiroshi Matsuoka, Hironobu Minami, Hiroshi Yokozaki, Markus G. Manz, Takashi Matozaki

**Affiliations:** ^1^ Division of Molecular and Cellular Signaling, Department of Biochemistry and Molecular Biology, Kobe University Graduate School of Medicine, Kobe, Japan; ^2^ Division of Biosignal Regulation, Department of Biochemistry and Molecular Biology, Kobe University Graduate School of Medicine, Kobe, Japan; ^3^ Division of Medical Oncology and Hematology, Department of Medicine, Kobe University Graduate School of Medicine, Kobe, Japan; ^4^ Division of Pathology, Department of Pathology, Kobe University Graduate School of Medicine, Kobe, Japan; ^5^ Division of Bioresource Research and Development, Department of Social/Community Medicine and Health Science, Kobe University Graduate School of Medicine, Kobe, Japan; ^6^ Department of Medical Oncology and Hematology, University and University Hospital Zurich, Zurich, Switzerland; ^7^ Comprehensive Cancer Center Zurich at the University of Zurich, Zurich, Switzerland

**Keywords:** humanized mouse, tumor-associated macrophages, SIRP alpha, cancer immunotherapy, patient-derived xenograft (PDX) model, macrophage checkpoint, B cell lymphoma

## Abstract

Tumor-associated macrophages (TAMs) are abundant in the tumor microenvironment and are considered potential targets for cancer immunotherapy. To examine the antitumor effects of agents targeting human TAMs *in vivo*, we here established preclinical tumor xenograft models based on immunodeficient mice that express multiple human cytokines and have been reconstituted with a human immune system by transplantation of human CD34^+^ hematopoietic stem and progenitor cells (HIS-MITRG mice). HIS-MITRG mice supported the growth of both human cell line (Raji)– and patient-derived B cell lymphoma as well as the infiltration of human macrophages into their tumors. We examined the potential antitumor action of an antibody to human SIRPα (SE12C3) that inhibits the interaction of CD47 on tumor cells with SIRPα on human macrophages and thereby promotes Fcγ receptor–mediated phagocytosis of the former cells by the latter. Treatment with the combination of rituximab (antibody to human CD20) and SE12C3 inhibited Raji tumor growth in HIS-MITRG mice to a markedly greater extent than did rituximab monotherapy. This enhanced antitumor effect was dependent on human macrophages and attributable to enhanced rituximab-dependent phagocytosis of lymphoma cells by human macrophages. Treatment with rituximab and SE12C3 also induced reprogramming of human TAMs toward a proinflammatory phenotype. Furthermore, the combination treatment essentially prevented the growth of patient-derived diffuse large B cell lymphoma in HIS-MITRG mice. Our findings thus support the study of HIS-MITRG mice as a model for the preclinical evaluation *in vivo* of potential therapeutics, such as antibodies to human SIRPα, that target human TAMs.

## Graphical Abstract

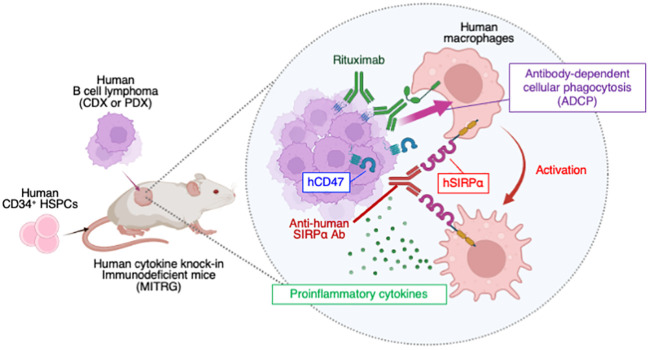

## 1 Introduction

The tumor microenvironment (TME) consists of tissue-resident cells and a large population of recruited immune cells. The immune component of the TME is thought to support tumor progression while limiting antitumor immune responses (1, 2). Tumor-associated macrophages (TAMs) are the most abundant infiltrating immune cell type in the TME of many human cancers (3). TAMs are associated with poor prognosis and manifest an anti-inflammatory phenotype that promotes angiogenesis, matrix remodeling, tumor growth, and metastasis (4, 5). However, TAMs also possess antitumor potential dependent on cytotoxicity for and phagocytosis of tumor cells (6). Reprogramming of TAMs from a protumoral state toward an antitumoral state might therefore provide the basis for new cancer therapeutics (7).

Signal regulatory protein α (SIRPα) is an immunoglobulin (Ig) superfamily protein that is highly expressed on macrophages and dendritic cells (8–11). Interaction of the extracellular region of SIRPα with its ligand CD47 elicits signaling by the tyrosine phosphatase SHP-1 that inhibits Fcγ receptor–mediated antibody-dependent cellular phagocytosis (ADCP). Interaction of CD47 on tumor cells with SIRPα on TAMs thus constitutes an innate immune checkpoint that interferes with the detection and phagocytosis of the former cells by the latter (10, 12). We previously showed that an antibody to SIRPα that blocks its interaction with CD47 markedly enhanced the inhibitory effect of rituximab, an antibody to human (h) CD20, on the growth of human B cell lymphoma in immunodeficient mice (13, 14). Given that these *in vivo* studies targeted endogenous mouse macrophages in the immunodeficient animals, however, a preclinical model that recapitulates the status of human TAMs in the TME is required to evaluate further the therapeutic effect of antibodies to hSIRPα (anti-hSIRPα) before their potential clinical use.

Humanized immune system (HIS) mice are immunodeficient mice reconstituted with a human immune system by transfer of human CD34^+^ hematopoietic stem and progenitor cells (HSPCs) (15–18). Recent advances in the development of HIS mice have allowed studies of the interaction between human immune components and human tumors (19, 20). In particular, HIS mouse models are essential for evaluation of the antitumor effects of therapeutics that act only on human immune cells, such as antibodies to the immune checkpoint molecule PD-1 (21). However, evaluation of the effects of tumor immunotherapeutic agents that target human macrophages (such as anti-hSIRPα) has not been possible with such models because the differentiation of human myeloid cells is not supported in immunodeficient mouse strains such as NOD *scid Il2rg* ^–/–^ (NSG) or NOD Shi*-scid Il2rg* ^null^ (NOG) (16). To overcome this problem, we previously generated MISTRG mice (with “S” referring to transgenic expression of hSIRPα), which support human myeloid cell development as a result of the knock-in replacement of mouse cytokine genes with their human orthologs (22, 23). HIS-MISTRG mice indeed support the functional differentiation of human TAMs in the TME, as demonstrated with a human melanoma xenograft model (22, 24).

To evaluate the antitumor response mediated by human TAMs subjected to anti-hSIRPα treatment *in vivo*, we have now developed human cell line– or patient-derived B cell lymphoma xenograft models in HIS-MITRG mice. We here demonstrate a TAM-dependent antitumor effect of anti-hSIRPα administered together with rituximab. Such treatment appeared to initiate the reprogramming of TAMs from a protumoral toward an antitumoral state. These models are thus suitable for the evaluation of TAM status as well as of antitumor therapy targeting TAMs *in vivo*.

## 2 Materials and methods

### 2.1 Mice

MITRG (*CSF1* ^h/h^ *IL3/CSF2* ^h/h^ *THPO* ^h/h^ *Rag2* ^–/–^ *Il2rg* ^–/–^) and MISTRG (*CSF1* ^h/h^ *IL3/CSF2* ^h/h^ *SIRPA* ^tg^ *THPO* ^h/h^ *Rag2* ^–/–^ *Il2rg* ^–/–^) mice were generated by Regeneron Pharmaceuticals as described previously (22). In these mice, mouse cytokine genes (*Csf1*, *Il3/Csf2*, *Thpo*) have been replaced by their human orthologs from the ATG start codon to the stop codon on a *Rag2* ^–/–^ *Il2rg* ^–/–^ double-knockout 129×Balb/c (N2) background with the use of VelociGene technology (25). MISTRG and MITRG mice were studied here under Material Transfer Agreement with Regeneron Pharmaceuticals. They were obtained from University Hospital Zurich and rederived by embryo transfer at CARD (Kumamoto University). The mice were bred and maintained in a room with restricted access at the Institute of Experimental Animal Research, Kobe University Graduate School of Medicine, under specific pathogen–free conditions, and with continuous prophylactic administration of enrofloxacin (Baytril, 0.27 mg/ml in drinking water). All animal experiments were approved by the Kobe University Animal Experimentation Regulatory Committee (permits P140508-R1, P140905, and P150204).

### 2.2 Human samples

Fresh umbilical cord blood (UCB) cells were obtained from Hyogo Cord Blood Cell Bank with written informed consent from parents. The use of UCB was approved by the ethics boards of Kobe University Graduate School of Medicine (approval no. 1820) and Hyogo Cord Blood Cell Bank (approval no. 201503). Diffuse large B cell lymphoma (DLBCL) specimens were obtained from Kobe University Hospital with written informed patient consent according to a protocol approved by the ethics board of Kobe University Graduate School of Medicine (approval no. 170144). Specimen information was anonymized and listed in a database.

### 2.3 Antibodies and reagents

Mouse monoclonal antibodies (mAbs) to hSIRPα [SE12C3 (IgG1) and 040 (IgG1)] were generated and purified as described previously (13, 26). 040 was further biotinated using Biotin Labeling Kit-NH_2_ (Dojindo, Japan). Anti-hCD47 mAbs (mIgG1) were generated from B6H12 hybridoma cells (ATCC). Rituximab was obtained from Chugai Pharmaceutical, and control mouse (m) IgG from Jackson ImmunoResearch. Peripheral blood (PB), splenocytes, bone marrow (BM), thymus, and tumor tissue of mice were tested in different panels for hCD45 (clone HI30), hCD19 (HIB19), hCD33 (WM53), hCD3 (HIT3a or OKT3), hCD4 (OKT4), hCD8 (HIT8a), hNKp46 (9E2), hCD14 (M5E2), CD11b (ICRF44 or M1/70), HLA-DR (L243), hCD1c (L161), hSIRPα/β (SE5A5), mCD45 (30-F11), mTER119 (TER119), and mF4/80 (BM8) (**Supplementary Table S1**). Alexa Fluor® 488 AffiniPure Goat Anti-Mouse IgG (H+L) secondary antibodies were purchased from Jackson ImmunoResearch, Streptavidin-Allophycocyanin (APC) and Zombie Aqua was obtained from BioLegend. Carboxyfluorescein diacetate succinimidyl ester (CFSE) was obtained from Thermo Fisher Scientific, and recombinant mouse and human macrophage colony-stimulating factor (M-CSF) from PeproTech.

### 2.4 Cell preparation from mice and flow cytometry

PB was collected from the retroorbital vein into heparin glass tubes. Leukocytes were prepared from PB, thymus, spleen, and BM as described previously (23). Tumors were minced into small pieces and incubated for 30 min at 37°C with Liberase TL and Liberase DL (50 μg/ml, Roche) or with collagenase IV (1 mg/ml, Worthington Biochemical) and DNase I (40 mg/ml, Sigma-Aldrich) in RPMI 1640 medium. The digest was passed through a 100-μm filter and treated with ammonium chloride–potassium (ACK) lysis buffer to eliminate red blood cells. The resulting single-cell suspensions were incubated with human TruStain FcX™ and anti-mCD16/32 (Biolegend), subsequently stained with fluorescently labeled antibodies, and then subjected to flow cytometric analysis with an LSRFortessa instrument (BD Biosciences). Data were analyzed with FlowJo X software version 10.9 (BD Biosciences).

### 2.5 Isolation of CD34^+^ cells from UCB mononuclear cells

Human CD34^+^ cells were purified from UCB mononuclear cells by density gradient centrifugation with Lymphoprep (Axis-shield) followed by positive immunomagnetic selection with the use of a CD34 MicroBead Kit UltraPure and LS columns (Miltenyi Biotec). Cells with a CD34^+^ purity of ≥95% and T cell contamination of <0.1% were used for transplantation in order to avoid xenogeneic graft versus host disease. Isolated CD34^+^ cells were either directly transplanted into mice or frozen in FBS containing 10% dimethyl sulfoxide and maintained at –80°C until use.

### 2.6 Generation of HIS mice

Recipient mice were engrafted with human HSPCs as previously described (22, 23), with minor modifications. In brief, newborn (up to 3 days of age) mice were subjected to sublethal (1.2 Gy) x-irradiation with a Hitachi MBR-1505R2 instrument before transplantation. CD34^+^ UCB cells (30,000 to 50,000) suspended in 20 to 25 μl of PBS were injected intrahepatically with a 26-gauge needle (Hamilton). Four to six weeks after injection, the percentage of hCD45^+^ cells among total (h+m) CD45^+^ cells in PB was measured by flow cytometry. Mice with >5% engraftment in PB were selected for *in vivo* experiments.

### 2.7 Development of PDX models

A renal subcapsular patient-derived xenograft (PDX) model of DLBCL was generated as previously described (27), with minor modifications. In brief, tumor tissue was obtained from DLBCL patients during surgery and was processed within 24h. Each fresh tumor specimen was cut into pieces of 2 by 2 by 2 mm and maintained in sterile RPMI 1640 medium until xenotransplantation. Mouse surgery was performed in a laminar-flow hood under sterile conditions. Introduction of an ~1-cm incision into the left flank of an anesthetized MITRG mouse was followed by incision of the peritoneum and exteriorization of the left kidney. A small pocket was created between the kidney capsule and parenchyma with the use of sharp tweezers and a stereomicroscope. A piece of tumor tissue was inserted into the pocket, the kidney was gently eased back into the peritoneal cavity, the peritoneal incision was aligned with 5-0 surgical threads, and the skin incision was fixed with a surgical stapler. Mice were maintained for 4 to 8 weeks until the tumor was palpable, after which they were killed, the tumor (P0) was excised and digested as described above for flow cytometry, and the isolated cells were stored at –80°C until use.

### 2.8 Tumor cell engraftment and treatment

Cell line–derived xenograft (CDX) models of B cell lymphoma were established as described previously (13, 14), with minor modifications. In brief, Raji or Raji^GFP/Luc^ cells (5 × 10^5^ in 100 μl of PBS) were injected subcutaneously into the right flank of HIS-MITRG or MITRG mice at 6 to 8 weeks of age. The mice were injected intraperitoneally with control mIgG (200 µg), rituximab (200 µg), SE12C3 (200 µg), rituximab (200 µg) plus SE12C3 (200 µg), or rituximab (200 µg) plus B6H12 (200 µg) three times per week beginning after the tumor volume had achieved an average of 60 mm^3^ in each treatment group. For PDX models of DLBCL, stored P0 tumor cells were thawed and washed with Iscove’s modified Dulbecco’s medium (IMDM) supplemented with 10% FBS and DNase I (50 µg/ml). The cells were suspended in PBS (1 × 10^6^ cells in 100 μl) and were injected subcutaneously into the right flank of HIS-MITRG or MITRG mice at 8 to 10 weeks of age. The mice were injected intraperitoneally with control mIgG (25 µg), rituximab (25 µg), or rituximab (25 µg) plus SE12C3 (25 µg) three times per week beginning after the tumor volume had achieved an average of 100 mm^3^ in each treatment group. Tumors were measured with digital calipers, and tumor volume was calculated as *a* × *b* ^2^/2, where *a* is the largest diameter and *b* is the smallest diameter.

### 2.9 Generation of Raji^GFP/LUC^ cells

Raji cells (ATCC) were engineered to express luciferase (Luc) and green fluorescent protein (GFP) with the use of the bidirectional lentiviral vector pSMAL-Luc (28). In brief, lentiviruses were generated by transfection of 293T cells with pSMAL-Luc together with the envelope plasmid pCAG-VSVG (Addgene) and the packaging plasmid psPAX2 (Addgene) and with the use of the Lipofectamine 2000 reagent (Life Technologies). The viruses were concentrated with the use of a Lenti-X concentrator (Clontech) and stored at –80°C until use. Raji cells (1.0 × 10^6^/ml) were infected with the lentiviruses at a multiplicity of infection of 5 for 24 h. The cells were then washed and cultured without virus for 5 days. GFP^+^ cells were sorted with the use of a FACSAriaIII instrument (BD Biosciences) in single-cell sorting mode, and several selected GFP^+^ colonies were tested for luciferase activity with the use of IVIS Spectrum system (PerkinElmer).

### 2.10 *In vivo* imaging

For whole-body bioluminescence imaging of mice engrafted with Raji^GFP/Luc^ cells, the animals were injected intraperitoneally with D-luciferin (VivoGlo Luciferin, Promega) at 150 mg/kg and analyzed 10 min later with an IVIS Spectrum system. Bioluminescence images were acquired with the “auto” setting and with F/stop = 1 and binning = medium. A digital false-color photon emission image of each mouse was generated, and photons were counted within the whole-body area. Photon emission was measured as radiance in photons s^–1^ cm^–2^ sr^–1^. Peak total flux values were assessed from the anatomic region of interest with the use of Living Image 4.0 (PerkinElmer) and were used for analysis.

### 2.11 Depletion of macrophages *in vivo*

For the depletion of macrophages *in vivo*, mice were injected intravenously with 200 μl of PBS-containing liposomes (control) or clodronate-containing liposomes (Liposoma B.V.) every 3 days for a total of five times starting 2 days before antibody treatment. The effectiveness of macrophage depletion was determined by flow cytometric analysis of hCD45^+^hCD14^+^ cells among BM cells or tumor cells of the treated animals.

### 2.12 *In vitro* cell culture and ADCP

For mouse macrophage culture, BM cells were isolated by crashing the tibia, femur, and spine of MITRG mice with a mortar and pestle. The cells were allowed to differentiate for at least 7 days by culture in IMDM supplemented with 10% FBS in the presence of recombinant murine M-CSF (16 ng/ml). For human macrophage culture, macrophages were generated by culture of UCB mononuclear cells in Iscove’s modified Dulbecco’s medium (IMDM) (Nacalai Tesque) supplemented with 10% FBS in the presence of recombinant human M-CSF (16 ng/ml). Mouse or human macrophages were then transferred to 24-well plates, to which were then added CFSE-labeled Raji cells together with control mIgG (10 μg/ml), rituximab (50 ng/ml), B6H12 (10 μg/ml), or SE12C3 (10 μg/ml). After incubation for 4 h at 37°C, phagocytosis of CFSE-labeled Raji cells by macrophages was quantified with an LSRFortessa instrument (BD Biosciences) as the percentage of CFSE-positive macrophages among total macrophages. To evaluate the expression of SIRPα after incubation with SE12C3 over time, UCB–derived human macrophages were incubated in the presence of control mIgG (1 μg/ml) or SE12C3 (1 μg/ml) for 2, 4, and 24h. Cells were collected after the indicated time points and stained with biotin anti-human SIRPα antibody (clone 040), followed by the staining with streptavidin-APC. The expression of SIRPα was measured by a MACSQuant analyzer10 instrument (Miltenyi Biotec).

### 2.13 Immunohistochemistry

Tumor tissue was fixed with 4% paraformaldehyde, dehydrated in ethanol, embedded in paraffin, and sectioned with a microtome. The sections were then depleted of paraffin, rehydrated in a graded series of ethanol solutions, and subjected to immunohistochemical staining for tumor-infiltrating macrophages with a rabbit mAb to hCD68 or mouse mAb to hCD163 antibodies at a dilution of 1:400 using the Leica BOND-MAX automated system and BOND Polymer Refine Detection Kit (Leica Biosystems). The stained sections were observed with a Keyence BZ-X700 microscope. The tissue area positive for staining was quantified in 20 randomly chosen sections per sample at a magnification of ×200 and with the use of BZ-X Analyzer 1.4.1.1 (Keyence). The positively stained areas were averaged for each sample.

### 2.14 Serum cytokine assays

Whole blood collected from HIS-MITRG mice was centrifuged for 15 min, and the serum supernatant was stored at –80°C until analysis. Multiple human cytokines in the serum samples were assayed with the use of a LEGENDplex Human Macrophage/Microglia Panel (13-plex) and a V-bottom Plate (BioLegend). Data were acquired with an LSRFortessa instrument (BD Biosciences) and analyzed with LEGENDplex Data Analysis software version 8.0 (BioLegend). Cytokine concentrations below the detection limit were assigned a value of 0.01 pg/ml.

### 2.15 Sorting of tumor-infiltrating macrophages

For sorting of TAMs, single-cell suspensions prepared from Raji^GFP/Luc^ cell–derived tumors of HIS-MITRG mice 14 days after the onset of antibody treatment were incubated first with biotin-labeled antibodies to hCD19 and to mCD45 and then with anti-biotin microbeads (Miltenyi Biotec). Biotin-labeled cells were depleted with the use of an autoMACS Pro Separator (Miltenyi Biotec), and the remaining cells were stained further for sorting of tumor-infiltrating macrophages as hCD45^+^HLA-DR^+^hCD14^+^hCD1c^–^ cells with the use of a FACSAriaIII instrument (BD Biosciences). The purity of the isolated cells was >98% as determined by flow cytometry (see **Supplementary Figure S5A**).

### 2.16 Preparation of cDNA and RT-qPCR analysis

Total RNA was extracted from freshly isolated TAMs with the use of an RNeasy Micro Kit (Qiagen). The RNA was subjected to reverse transcription (RT) with the use of a QuantiTect Reverse Transcription Kit (Qiagen), and the resulting cDNA was subjected to quantitative PCR (qPCR) analysis in 384-well plates (Roche) with the use of a QuantiTect SYBR Green PCR Kit (Qiagen) and a LightCycler 480 instrument (Roche). The abundance of each target mRNA was normalized by that of *ACTB* mRNA. Primer sequences for qPCR analysis are provided in **Supplementary Table S2**.

### 2.17 RNA-seq analysis of tumor-infiltrating macrophages

Total RNA was extracted from sorted hCD14^+^ TAMs of Raji^GFP/Luc^ cell–derived tumors from HIS-MITRG mice with the use of an RNeasy Micro Kit (Qiagen). The RNA was subjected to RT, and the resulting cDNA was amplified with the use of a SMART-Seq V4 Ultra Low Input RNA Kit for Sequencing (Clontech). RNA–sequencing (seq) libraries were prepared with a Nextera XT DNA Library Preparation Kit (Illumina). Single-end 75-bp sequencing was performed with an Illumina HiSeqX system. RNA-seq reads were aligned to the human reference genome (GRCh38) with the use of the STAR aligner (29). Raw read counts were obtained with RSEM and were normalized for further analysis with the built-in normalization algorithms of DESeq2 (30). Differential expression genes (DEGs) were defined as with base means >500, an adjusted *P* value of <0.05, and a log_2_[fold change] of >2 for comparison between any two groups. A heat map was generated by the Bioconductor package Pheatmap. Gene set enrichment analysis (GSEA) (http://www.broad.mit.edu/gsea) was used to calculate the enrichment of genes in hallmark gene sets of the Molecular Signatures Database (MSigDB) v7.3. It was performed with a list of genes ranked according to their differential expression between two groups with the use of the GSEA preranked function in Gsea-4.1 software.

### 2.18 Statistical analysis

Data are presented as means ± SEM, unless indicated otherwise, and were analyzed by Student’s *t* test; one-way ANOVA followed by Tukey’s test; Welch’s ANOVA; or two-way repeated-measures ANOVA followed by Šídák’s test with the use of Prism9 software version 9.4.1 (GraphPad Software). A *P* value of <0.05 was considered statistically significant.

## 3 Results

### 3.1 Human immune cells promote tumor growth in HIS-MITRG mice

**Figure 1:**
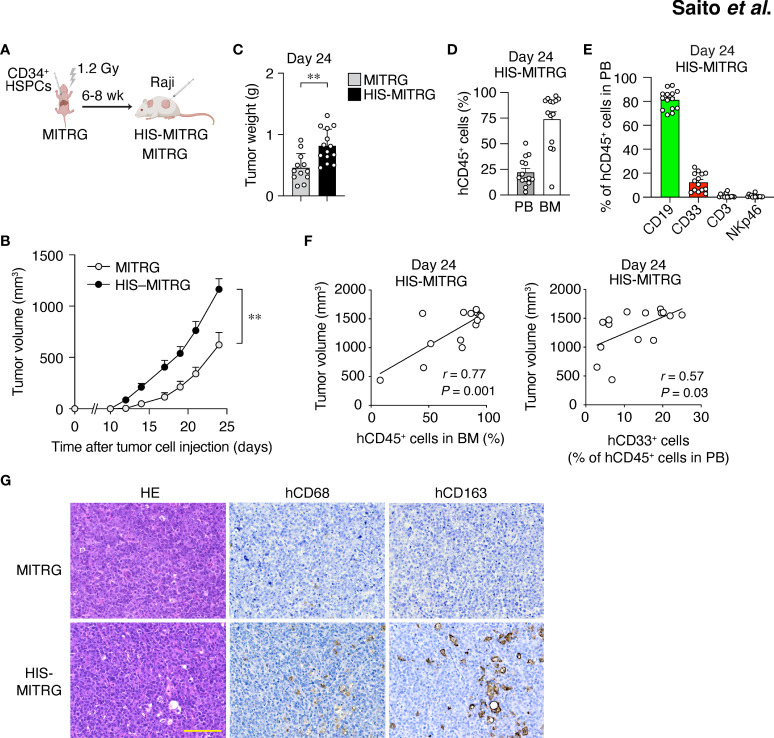
Human immune cells promote tumor growth in HIS-MITRG mice. **(A)** Newborn MITRG mice were subjected to sublethal irradiation followed by intrahepatic injection of human UCB-derived CD34^+^ cells. Raji cells were injected subcutaneously into the flank of the resulting HIS-MITRG mice at 6 to 8 weeks (wk) after hCD34^+^ cell transplantation. **(B)** Tumor volume was determined at the indicated times after Raji cell injection in HIS-MITRG or control MITRG mice. **(C)** Tumor weight in HIS-MITRG or MITRG mice at 24 days after Raji cell injection. **(D)** Percentage of hCD45^+^ cells among total (human and mouse) CD45^+^ cells in PB and BM of HIS-MITRG mice at 24 days after Raji cell injection. **(E)** Frequency of CD19^+^, CD33^+^, CD3^+^, or NKp46^+^ cells among hCD45^+^ cells in PB of HIS-MITRG mice at 24 days after Raji cell injection. Data in **(B)** to **(E)** are means + SEM for *n* = 12 (MITRG) or *n* = 14 (HIS-MITRG) mice examined in four experiments. **P < 0.01 [two-way repeated-measures ANOVA followed by Šídák’s test **(B)**, or Student’s *t* test **(C)**]. **(F)** Spearman correlation analysis for tumor volume versus the percentage of hCD45^+^ cells in BM or the percentage of hCD33^+^ cells among hCD45^+^ cells in PB of HIS-MITRG mice at 24 days after Raji cell injection. **(G)** Hematoxylin-eosin (HE) staining as well as hCD68 and hCD163 immunohistochemical staining of tumor tissue from MITRG or HIS-MITRG mice at 24 days after Raji cell injection. Images are representative of three (MITRG) or five (HIS-MITRG) mice. Scale bar, 100 μm.

We previously generated a strain of immunodeficient mice (designated MITRG) in which the genes encoding M-CSF (CSF1), interleukin-3 (IL-3) and granulocyte-macrophage colony-stimulating factor (GM-CSF or CSF2), and thrombopoietin (THPO) are replaced by their human orthologs (22). To generate HIS-MITRG mice, we subjected newborn MITRG mice (up to 3 days after birth) to sublethal (1.2 Gy) irradiation followed by intrahepatic injection of human CD34^+^ HSPCs isolated from UCB (**Figure 1A**). At 6 to 8 weeks after transplantation, the percentage of hCD45^+^ cells among total (human and mouse) CD45^+^ cells in PB was measured by flow cytometry (23). Given that the reconstitution efficiency of the mice varied, we selected animals in which the proportion of hCD45^+^ cells in blood was >5% for subsequent experiments (**Supplementary Figure S1A**).

To examine the impact of differentiated human immune cells on tumor growth in HIS-MITRG mice, we subcutaneously injected the mice with human B cell lymphoma Raji cells (500,000 cells/mouse) at 6 to 8 weeks of age. Compared with similarly injected MITRG mice, the HIS-MITRG mice manifested a significantly greater tumor growth rate and tumor weight (**Figures 1B, C** and **Supplementary Figure S1B**). Substantial engraftment of hCD45^+^ cells was apparent in BM as well as in PB of HIS-MITRG mice at 24 days after tumor cell injection (**Figure 1D**). Among hCD45^+^ cells in PB, hCD19^+^ B cells were predominant at this time (**Figure 1E**). Tumor volume at 24 days was significantly correlated with the percentage of hCD45^+^ cells among BM cells as well as with the frequency of hCD33^+^ myeloid cells in PB (**Figure 1F**). Histological analysis of tumor tissue from HIS-MITRG mice revealed infiltration of hCD68^+^ macrophages—in particular, hCD163^+^ macrophages—with a signature starry-sky appearance characterized by engulfment of apoptotic tumor cells by the TAMs (**Figure 1G**). To examine the effect of transgenic expression of hSIRPα in endogenous mouse macrophages on tumor growth in HIS-MITRG mice, we compared HIS-MISTRG and HIS-MITRG mice in this context. The tumor growth rate and tumor weight at day 24 after tumor cell engraftment were both similar in HIS-MISTRG and HIS-MITRG mice (**Supplementary Figures S1C, D**). Human lymphoma growth was thus promoted in HIS-MITRG mice compared with MITRG mice, likely as a result of the engraftment of human myeloid cells including macrophages.

### 3.2 The hSIRPα Ab SE12C3 enhances rituximab-induced inhibition of B cell lymphoma growth in HIS-MITRG mice

**Figure 2:**
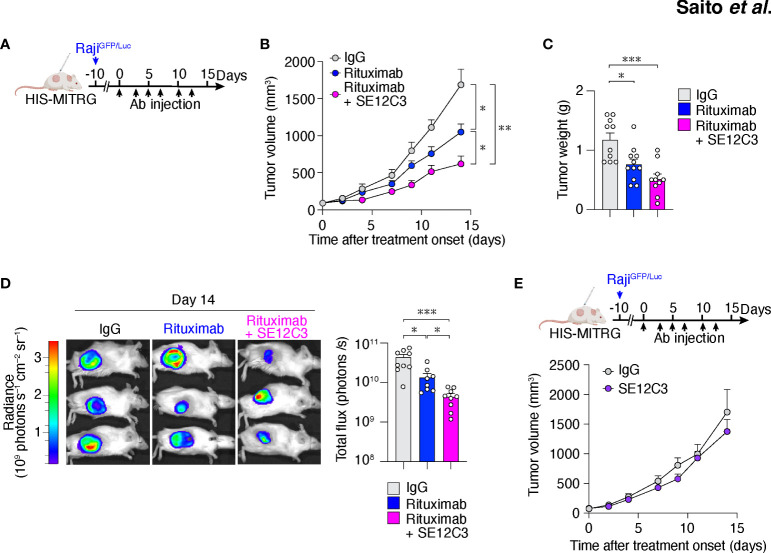
The hSIRPα antibody SE12C3 enhances rituximab-induced inhibition of B cell lymphoma growth in HIS-MITRG mice. **(A)** HIS-MITRG mice at 6 to 8 weeks of age were injected subcutaneously with Raji^GFP/Luc^ cells, and were treated intraperitoneally with normal mIgG, rituximab, or both rituximab and SE12C3 three times per week beginning when the tumors in each group had achieved an average volume of 60 mm^3^. **(B)** Tumor volume was determined at the indicated times after treatment onset for mice as in **(A)**. **(C)** Tumor weight at 14 days after treatment onset for mice as in **(A)**. Data in **(B, C)** are means + SEM for *n* = 10 (control IgG or rituximab + SE12C3) or *n* = 11 (rituximab) mice examined in five experiments. **(D)** Bioluminescence imaging of tumors was performed at 14 days after treatment onset for mice as in **(A)**. Representative images and quantitative data for peak total flux (means + SEM) are shown for *n* = 9 (control IgG or rituximab + SE12C3) or *n* = 8 (rituximab) mice examined in three experiments. **(E)** HIS-MITRG mice harboring Raji^GFP/Luc^ tumors were treated with control IgG (*n* = 8) or SE12C3 (*n* = 8) as in **(A)**, and tumor volume was determined at the indicated time points after treatment onset. Data are means + SEM for mice examined in two experiments. *P < 0.05, **P < 0.01, ***P < 0.001 [two-way repeated-measures ANOVA followed by Šídák’s test **(B, E)**; one-way ANOVA followed by Tukey’s test **(C)**; or Welch’s ANOVA **(D)**].

We next examined whether anti-hSIRPα might enhance the antitumor effect of rituximab *in vivo*. To avoid the binding of anti-hSIRPα to hSIRPα expressed on mouse macrophages, we performed the following experiments with HIS-MITRG mice rather than HIS-MISTRG mice. Raji tumor cells express pro-phagocytic molecules such as calreticulin together with CD47, and the interaction of hCD47 on Raji cells with hSIRPα on human macrophages likely inhibits phagocytosis of tumor cells (31). hSIRPα is indeed expressed on human CD14^+^ macrophages in the bone marrow and tumor of HIS-MITRG mice, whereas it is not expressed on mouse F4/80^+^ macrophages (**Supplementary Figure S2A**). HIS-MITRG mice that had been injected with Raji cells expressing GFP and firefly luciferase (Raji^GFP/Luc^ cells) were treated intraperitoneally three times per week with control mIgG, rituximab, or rituximab plus the hSIRPα antibody SE12C3 beginning when the tumors had achieved an average volume of 60 mm^3^ (**Figure 2A**). SE12C3 blocks the interaction of hCD47 with hSIRPα by binding to the NH_2_-terminal IgV domain of hSIRPα, but it does not bind to mSIRPα (13). We confirmed that SE12C3 specifically reacts with human macrophages, but not mouse macrophages (**Supplementary Figure S2B**). In addition, the expression of hSIRPα on human macrophages was not reduced up to 24h in the presence of SE12C3 compared with control IgG (**Supplementary Figure S2C**). Thus, SE12C3 specifically binds to hSIRPα and does not affect the expression of hSIRPα on human macrophages.

Treatment with rituximab significantly inhibited Raji^GFP/Luc^ tumor growth in HIS-MITRG mice compared with control IgG treatment. Moreover, tumor growth in mice treated with the combination of rituximab and SE12C3 was significantly inhibited compared with that apparent in mice of the other two groups (**Figure 2B**; **Supplementary Figure S2D**). Tumor weight was significantly smaller in HIS-MITRG mice treated with rituximab or with rituximab and SE12C3 than in those treated with control IgG (**Figure 2C**). *In vivo* bioluminescence imaging of tumors also revealed a significant reduction in tumor volume in mice treated with rituximab and SE12C3 compared with those treated with control IgG or with rituximab (**Figure 2D**). By contrast, treatment with SE12C3 alone had no effect on tumor volume (**Figure 2E**). These results thus indicated that SE12C3 enhanced the antitumor effect of rituximab in HIS-MITRG mice. Furthermore, such enhancement was not apparent in MITRG mice (**Supplementary Figure S2E**), suggesting that it was dependent on engrafted human immune cells.

### 3.3 Treatment with rituximab alone or together with SE12C3 promotes infiltration of human macrophages in tumors of HIS-MITRG mice

To explore whether reconstituted human immune cell subsets contribute to the enhanced inhibition of tumor growth apparent in HIS-MITRG mice treated with the combination of rituximab and SE12C3, we characterized human immune cells in tumor-bearing HIS-MITRG mice at 14 days after the onset of antibody treatment. The frequency of hCD45^+^ cells in BM, PB, and the spleen of tumor-bearing HIS-MITRG mice was lower for those treated with rituximab either alone or together with SE12C3 than for those treated with control mIgG (**Supplementary Figure S3A**). Among hCD45^+^ cells, the proportions of different immune cell types were altered by treatment with rituximab or with rituximab plus SE12C3, with a marked reduction in the percentage of hCD19^+^ B cells in both BM and PB being apparent (**Supplementary Figure S3B**). By contrast, the frequency of hCD45^+^ cells among thymocytes as well as that of thymocyte subsets did not differ substantially among the treatment groups (**Supplementary Figures S3A, C**). The reduction in the proportion of hCD45^+^ cells induced by treatment with rituximab in HIS-MITRG mice was therefore largely attributable to a reduction in the number of human B cells.

**Figure 3:**
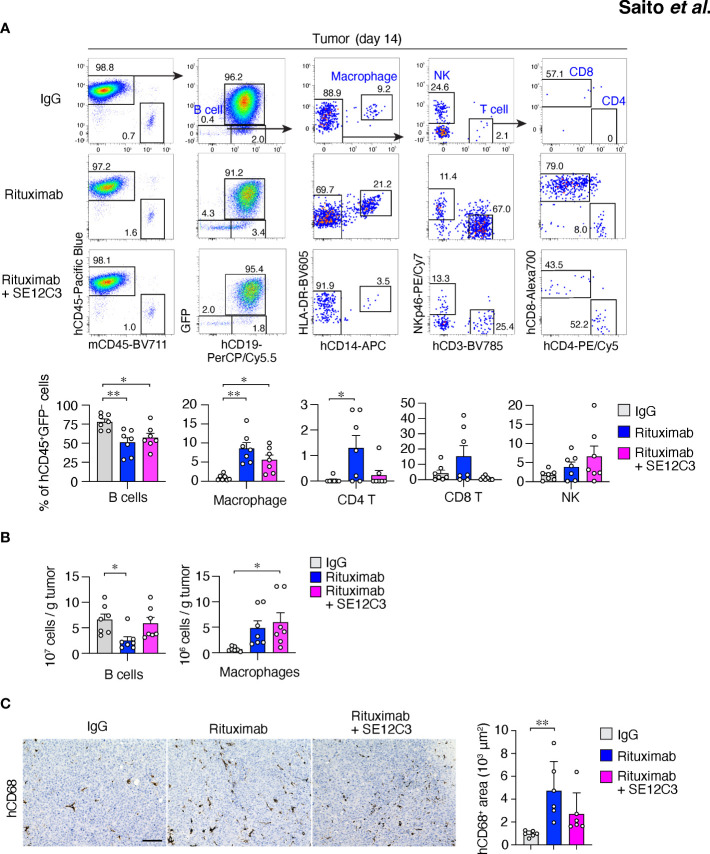
Treatment with rituximab or with rituximab plus SE12C3 increases the number of human macrophages in tumors of HIS-MITRG mice. **(A)** Representative FACS plots for human immune cells in Raji^GFP/Luc^ tumors of HIS-MITRG mice at 14 days after the onset of antibody treatment as in **Figure 2A**. The percentages of B cells (hCD19^+^ cells), macrophages (hCD19^–^hCD14^+^HLA-DR^+^ cells), CD4^+^ or CD8^+^ T cells (hCD19^–^hCD14^–^hCD3^+^hCD4^+^ or hCD8^+^cells), and natural killer (NK) cells (hCD19^–^hCD14^–^hNKp46^+^cells) among hCD45^+^GFP^–^ cells were also determined by flow cytometry and are shown below the FACS plots. **(B)** Numbers of tumor-infiltrating B cells or macrophages determined as in **(A)** and presented as cells per gram of tumor tissue. Quantitative data in **(A, B)** are means + SEM for *n* = 7 mice per group examined in four experiments. **(C)** Representative immunohistochemical staining of hCD68 in tumor tissue at 14 days after the onset of antibody treatment for mice as in **(A)**. Scale bar, 100 µm. The area positive for hCD68 staining in randomly selected sections was also measured and is shown as mean + SEM values for *n* = 6 mice per group. *P < 0.05, **P < 0.01(one-way ANOVA followed by Tukey’s test).

We next characterized tumor-infiltrating immune cells after antibody treatment. Among hCD45^+^GFP^–^ cells in Raji^GFP/Luc^ tumors of HIS-MITRG mice, the frequency of hCD19^+^ B cells was also significantly reduced by treatment with rituximab either alone or together with SE12C3 relative to treatment with control IgG (**Figure 3A**). In contrast, the frequency of HLA-DR^+^hCD14^+^ macrophages among hCD45^+^GFP^–^ cells was significantly increased by treatment with rituximab or with rituximab plus SE12C3 (**Figure 3A**). Combined treatment with rituximab and SE12C3 also significantly increased the number of HLA-DR^+^hCD14^+^ macrophages per gram of tumor tissue compared with control IgG treatment (**Figure 3B**). Consistent with these findings, immunohistochemical analysis revealed that the area occupied by hCD68^+^ macrophages in tumor tissue was increased by treatment with rituximab or with rituximab plus SE12C3 (**Figure 3C**). Collectively, these results suggested that treatment with rituximab alone or together with SE12C3 promotes the infiltration of human macrophages into tumor tissue of HIS-MITRG mice.

### 3.4 Importance of human macrophages for inhibition of tumor growth by rituximab-SE12C3 in HIS-MITRG mice

**Figure 4:**
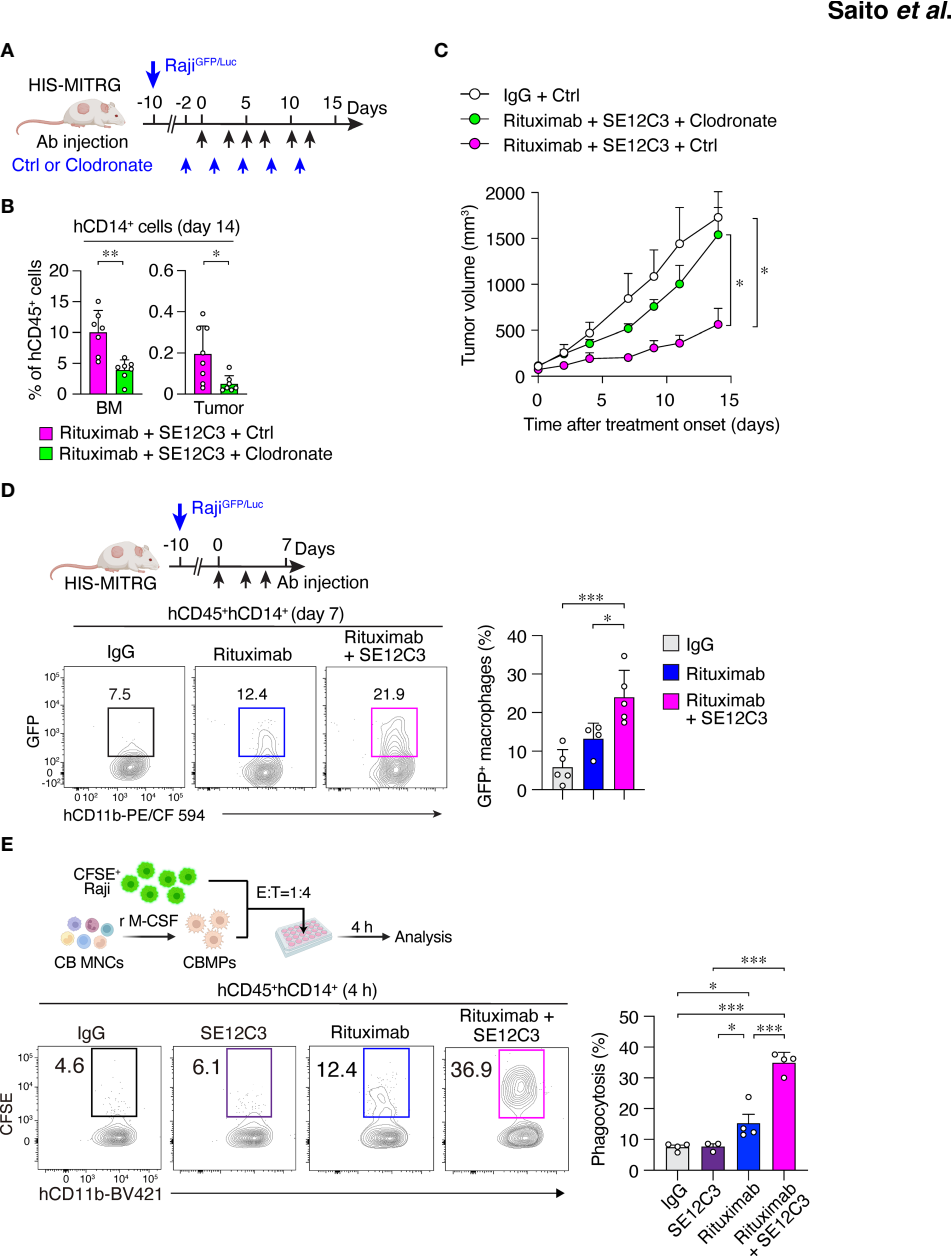
Importance of human macrophages for inhibition of tumor growth by SE12C3 and rituximab in HIS-MITRG mice. **(A)** HIS-MITRG mice bearing Raji^GFP/Luc^ tumors were injected intravenously with PBS (Ctrl) liposomes or clodronate liposomes beginning before the onset of antibody treatment according to the indicated schedule. **(B)** The percentage of hCD14^+^ cells among hCD45^+^ cells in BM and tumor tissue was determined by flow cytometry at 14 days after the onset of antibody treatment for mice as in **(A)**. **(C)** Time course of tumor volume for mice as in **(A)**. Data in **(B, C)** are means + SEM for *n* = 7 mice per group examined in three experiments. **(D)** HIS-MITRG mice harboring Raji^GFP/Luc^ tumors were treated with control mIgG (*n* = 5), rituximab (*n* = 4), or rituximab plus SE12C3 (*n* = 5) three times per week. The percentage of GFP^+^ cells among hCD45^+^hCD11b^+^hCD14^+^ cells in tumor tissue at 7 days after treatment onset was determined by flow cytometry. Representative plots and averaged data (means + SEM) are shown for mice examined in two experiments. **(E)** CFSE-labeled Raji cells were incubated for 4 h with macrophages (CBMPs) derived from human UCB mononuclear cells (CB MNCs) and in the presence of the indicated antibodies. The effector:target (E:T) ratio was 1:4, and the proportion of CFSE^+^CD11b^+^ cells among total CD11b^+^ cells was determined by flow cytometry as percentage phagocytosis as indicated. Representative results and averaged data (means + SEM) for three or four independent experiments are shown. *P < 0.05, **P < 0.01, ***P < 0.001 [Student’s *t* test **(B)**, two-way repeated-measures ANOVA followed by Šídák’s test **(C)**, or one-way ANOVA followed by Tukey’s test **(D, E)**].

We next examined whether macrophages contribute to the antitumor effect of combined treatment with SE12C3 and rituximab in HIS-MITRG mice. The mice were depleted of macrophages by repeated intravenous injection of clodronate-containing liposomes beginning 2 days before the onset of antibody treatment (**Figure 4A**). The percentage of hCD14^+^ cells among hCD45^+^ cells was significantly reduced in both BM and tumor tissue at 14 days after the onset of rituximab-SE12C3 treatment for mice injected with clodronate liposomes compared with those injected with control (PBS-containing) liposomes (**Figure 4B**; **Supplementary Figure S4A**). The antitumor effect of rituximab-SE12C3 was markedly attenuated in mice depleted of human macrophages (**Figure 4C**). To elucidate further the role of human macrophages in the elimination of tumor cells, we examined the incorporation of GFP in tumor-infiltrated human macrophages at 7 days after treatment onset in HIS-MITRG mice bearing Raji^GFP/Luc^ tumors (**Figure 4D**). Among tumor-infiltrating hCD45^+^hCD14^+^ cells, the frequency of GFP^+^ cells, which would be expected to reflect engulfment of GFP^+^ tumor cells by human macrophages, was significantly increased for HIS-MITRG mice treated with rituximab and SE12C3 compared with those treated with control mIgG or with rituximab alone (**Figure 4D**). We also examined the ADCP activity of human macrophages toward CFSE-labeled Raji cells *in vitro* (**Figure 4E**). SE12C3 significantly enhanced rituximab-dependent phagocytosis of CFSE-labeled Raji cells by human macrophages generated from UCB-derived mononuclear cells, whereas such phagocytosis activity was minimal in the presence of SE12C3 alone (**Figure 4E**). By contrast, whereas rituximab alone promoted the phagocytosis of Raji cells by mouse macrophages generated from MITRG mouse BM cells, SE12C3 did not enhance this effect of rituximab (**Supplementary Figure S4B**). These results thus suggested that the antitumor effect of SE12C3 in combination with rituximab is attributable to enhancement by SE12C3 of rituximab-dependent tumor cell phagocytosis by human macrophages.

### 3.5 SE12C3 and rituximab alter the phenotype of human tumor-infiltrating macrophages *in vivo*

**Figure 5:**
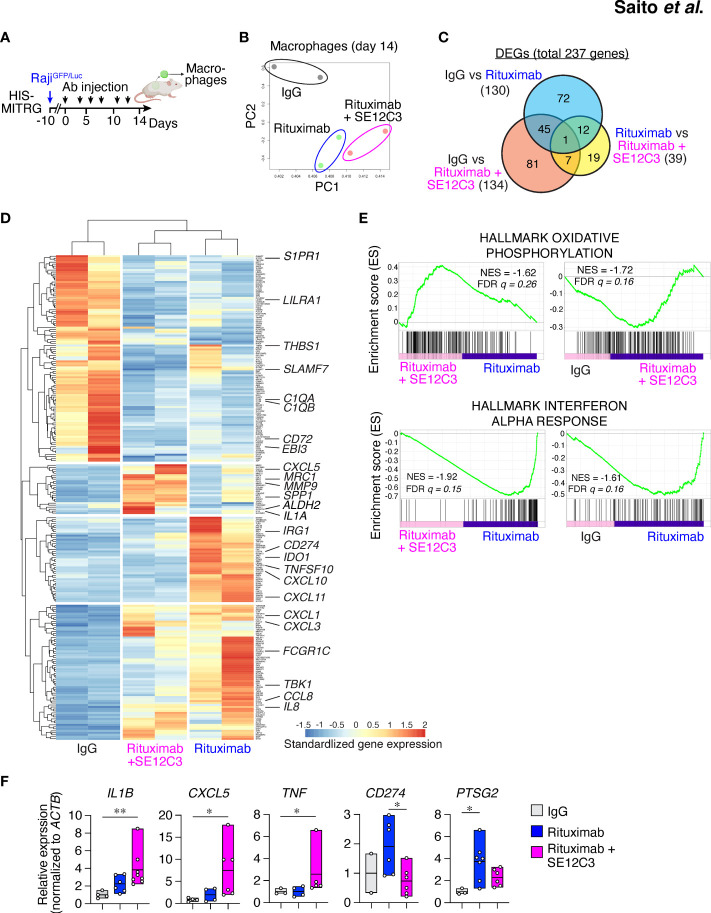
SE12C3 and rituximab alter the phenotype of human tumor-infiltrating macrophages *in vivo*. **(A)** HIS-MITRG mice were injected with Raji^GFP/Luc^ cells and subjected to antibody treatment, and human macrophages were sorted from the resulting tumors at 14 days after the onset of treatment for RNA-seq and RT-qPCR analyses. **(B)** Principal component (PC) analysis of all genes for RNA-seq analysis of the three treatment groups (*n* = 2 mice for each treatment group). **(C)** Venn diagram of DEGs identified by RNA-seq analysis. **(D)** Heat map analysis of the DEGs. **(E)**, GSEA for pairwise comparisons of IgG, rituximab, and rituximab-SE12C3 treatment groups. NES, normalized enrichment score; FDR, false discovery rate. **(F)** RT-qPCR analysis of gene expression in the tumor-infiltrating macrophages isolated from mice after treatment with control IgG (*n* = 2 to 4), rituximab (*n* = 4 to 7), or rituximab plus SE12C3 (*n* = 5 to 7). Data are presented as box plots indicating the median and upper and lower quartiles. *P < 0.05, **P < 0.01 (one-way ANOVA followed by Tukey’s test).

To characterize further the phenotype of TAMs in the TME after antibody treatment in HIS-MITRG mice, we performed RNA-seq analysis of hCD45^+^hCD14^+^HLA-DR^+^ cells sorted from Raji^GFP/Luc^ tumors of these mice treated with control IgG, rituximab, or rituximab plus SE12C3 (**Figure 5A**; **Supplementary Figure S5A**). Principal component analysis separated the IgG treatment group from the rituximab monotherapy and rituximab-SE12C3 combination therapy groups (**Figure 5B**). Out of 14,919 profiled transcripts, we identified 237 DEGs (**Figure 5C**). There were fewer DEGs for the rituximab monotherapy group versus the rituximab-SE12C3 group (39 genes) than for the IgG group versus either the rituximab group (130 genes) or the rituximab-SE12C3 group (134 genes). Heat map analysis revealed four clusters of DEGs (**Figure 5D**), one of which was characterized by genes whose expression was upregulated only in the rituximab-SE12C3 group and included *CXCL5* and *SPP1*, both of which are related to inflammatory TAMs (32). By contrast, a cluster of genes upregulated only in the rituximab monotherapy group included *CD274* (PD-L1) and *IDO1* (33, 34), and a cluster of genes upregulated in both rituximab and rituximab-SE12C3 groups included *CCL8* and *IL-8*. The final cluster containing genes upregulated only in the IgG group included genes associated with M2-like macrophages such as *C1QA*, *C1QB*, and *SLAMF7* (32). GSEA revealed that genes related to oxidative phosphorylation were upregulated after treatment with rituximab and SE12C3 compared with that with rituximab alone or control IgG (**Figure 5E**). By contrast, genes related to the interferon-α response were significantly upregulated in the rituximab group compared with the rituximab-SE12C3 or IgG groups. Furthermore, RT-qPCR analysis of sorted human tumor-infiltrating macrophages revealed that treatment with rituximab and SE12C3 resulted in significant upregulation of *IL1B*, *TNF*, and *CXCL5* expression compared with that apparent for the control IgG group, whereas rituximab monotherapy resulted in significant upregulation of *PTGS2* (COX2) and *CD274* (PD-L1) expression compared with the control IgG or rituximab-SE12C3 groups, respectively (**Figure 5F**; **Supplementary Figure S5B**). Together, these results suggested that treatment with rituximab alone or with rituximab together with SE12C3 alters the phenotype of human macrophages in the TME.

We further examined the concentration of human cytokines in serum obtained from Raji^GFP/Luc^ tumor–bearing HIS-MITRG mice at 14 days after the onset of antibody treatment. The concentration of TNF was significantly increased in mice treated with both rituximab and SE12C3 compared with those treated with control IgG (**Supplementary Figure S5C**). By contrast, the concentrations of inhibitory cytokines such as CCL17, IL-10, and IL-1RA were significantly lower in mice treated with rituximab and SE12C3 than in those treated with control IgG or with rituximab alone (**Supplementary Figure S5C**).

### 3.6 HIS-MITRG mice support human primary DLBCL engraftment and SE12C3 enhances the antitumor effect of rituximab in a PDX model

**Figure 6:**
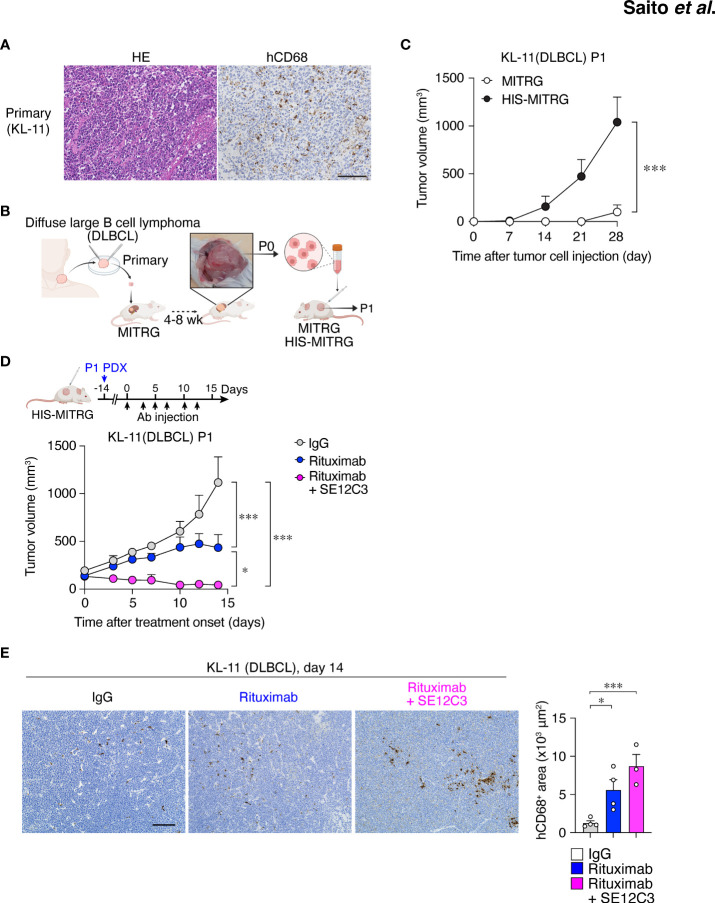
HIS-MITRG mice support primary DLBCL engraftment and SE12C3 enhances the antitumor effect of rituximab in such a PDX model. **(A)** Hematoxylin-eosin staining and immunohistochemical staining of hCD68 for tumor tissue from a patient with DLBCL. Scale bar, 100 μm. **(B)** Primary tumor tissue from a DLBCL patient was implanted under the kidney capsule of a MITRG mouse. The resulting tumor (P0) was isolated after 4 to 8 weeks, and the dissociated tumor cells were transplanted subcutaneously into HIS-MITRG or MITRG mice for monitoring of tumor (P1) growth. **(C)** Time course for the growth of P1 tumors in MITRG (*n* = 4) or HIS-MITRG (*n* = 3) mice at 6 to 8 weeks of age. Data are means + SEM for mice examined in two experiments. **(D)** HIS-MITRG mice bearing P1 tumors with an average volume of 100 mm^3^ were treated intraperitoneally with control mIgG, rituximab, or rituximab plus SE12C3 three times per week, and tumor volume was determined at the indicated times after treatment onset. **(E)** Representative immunohistochemical staining of hCD68 in P1 tumors isolated from HIS-MITRG mice at 14 days after the onset of antibody treatment. Scale bar, 100 μm. The area positive for hCD68 staining in randomly selected sections was also measured. Quantitative data in **(D, E)** are means + SEM for *n* = 4 mice (control IgG or rituximab) or *n* = 3 mice (rituximab + SE12C3) examined in two experiments. *P < 0.05, ***P < 0.001 [two-way repeated-measures ANOVA followed by Šídák’s test **(C, D)**, or one-way ANOVA followed by Tukey’s test **(E)**].

DLBCL is the most common type of non-Hodgkin lymphoma in adults, accounting for up to 35% of such cases (35). Immunohistochemical staining for hCD68 revealed infiltration of macrophages in primary DLBCL tumor tissue (**Figure 6A**). To establish a PDX model of DLBCL, we implanted primary tumor tissue from a patient with non–germinal center B cell–like DLBCL in the renal subcapsular space of a MITRG mouse (**Figure 6B**) (27). After 1 to 2 months, the resulting tumor (P0) was removed and dissociated for isolation of tumor cells (**Figure 6B**). Both primary and P0 tumor cells were monoclonal for the Igκ chain, indicating that only lymphoma cells were expanded in the initial host animal (**Figure S6A**). We next examined the impact of the engrafted human immune cells on PDX tumor growth in HIS-MITRG mice. The P0 tumor cells (1 × 10^6^ per mouse) were thus transplanted subcutaneously into adult HIS-MITRG or MITRG mice. Similar to the Raji xenograft model (**Figure 1B**), HIS-MITRG mice supported tumor growth to a significantly greater extent than did MITRG mice (**Figure 6C**; **Supplementary Figure S6B**). We then examined the effects of rituximab and SE12C3 treatment on DLBCL tumor growth in the HIS-MITRG mouse–based PDX model after tumors had achieved an average size of 100 mm^3^ (**Figure 6D**). Treatment with rituximab alone resulted in significant inhibition of tumor growth compared with that apparent in mice treated with control mIgG, and this antitumor effect was significantly enhanced by additional administration of SE12C3 (**Figure 6D**; **Supplementary Figure S6C**). The essentially complete inhibition of tumor growth apparent in mice treated with both rituximab and SE12C3 was achieved even though the amount of both antibodies was reduced to 25 μg per dose, compared with the 200 µg per dose used in the Raji–HIS-MITRG model. Moreover, immunohistochemical analysis of tumor tissue revealed that the extent of hCD68^+^ macrophage infiltration was markedly greater for mice treated with rituximab or with rituximab plus SE12C3 than for those treated with control IgG (**Figure 6E**). Together, these results thus indicated that SE12C3 enhanced the antitumor effect of rituximab in the PDX model of DLBCL in HIS-MITRG mice.

## 4 Discussion

With the use of both CDX and DLBCL PDX models, we have here demonstrated enhanced B cell lymphoma growth in HIS-MITRG mice compared with MITRG mice as well as tumor infiltration of human immune cells in the former mice. Such enhanced lymphoma growth correlated with the engraftment of human immune cells—in particular, myeloid cells—consistent with previous observations with HIS-MISTRG mice (22, 24, 36). Whereas the precise mechanism by which human myeloid cells promote lymphoma growth in HIS-MITRG mice remained unclear, we observed that the tumor-infiltrated macrophages in the CDX model expressed the M2-like macrophage marker CD163 at a high level. Moreover, human macrophages isolated from tumors of mice treated with control IgG showed upregulation of M2-like macrophage marker genes compared with those from mice treated with rituximab either alone or together with SE12C3. The infiltration of M2-like macrophages has previously been observed in human B cell lymphoma specimens (37). Our preclinical tumor models thus appear to recapitulate, at least in part, the protumorigenic role of TAMs in B cell lymphoma (38).

Blockade of the CD47-SIRPα interaction is a promising TAM-directed therapeutic approach, with the aim of promoting ADCP of tumor cells by macrophages (39, 40). SE12C3 binds to hSIRPα, but not to mSIRPα, and blocks the human CD47-SIRPα interaction (13). Indeed, we found that SE12C3 markedly enhanced phagocytosis of rituximab-opsonized Raji cells by human macrophages, whereas such an effect of SE12C3 was not apparent with mouse macrophages. Moreover, depletion of macrophages in HIS-MITRG mice resulted in attenuation of the antitumor effect of the combination of rituximab and SE12C3. Together, these observations indicate that human macrophages are largely responsible for the antitumor action of combination therapy with SE12C3 and rituximab in our HIS-MITRG preclinical tumor models.

The number of human tumor-infiltrated macrophages was increased by rituximab treatment for both CDX and PDX tumor models in HIS-MITRG mice. In addition, RNA-seq analysis revealed that these TAMs in the CDX model showed increased expression of IFN-regulated genes such as *CXCL10* and *CD274* (PD-L1) after treatment with rituximab (33, 41). Indeed, GSEA revealed the upregulation of genes related to the interferon-α response in these cells after rituximab treatment (42). Such macrophages are upregulated by IDO1 and IRG1 expression and are thought to limit inflammatory responses (43, 44). Together, these results suggest that rituximab treatment conferred an immunosuppressive phenotype on TAMs, likely as a result of the induction of ADCP (45).

Expression of *CD274* and *IDO1* was downregulated and that of a variety of genes related to proinflammatory responses was upregulated in TAMs from mice treated with both SE12C3 and rituximab compared with those from mice treated with rituximab alone. For example, we found that the expression of *CXCL5* was upregulated, and the expression of this gene was previously shown to be increased during efferocytosis of tumor cells (46). Moreover, the combination of SE12C3 and rituximab increased the expression of proinflammatory cytokine genes such as *IL1B* and *TNF* in TAMs as well as the concentration of TNF in serum. These results thus suggest that blockade of the CD47-SIRPα interaction induced the adoption of a proinflammatory phenotype in TAMs. Interaction of SE12C3 with the orphan receptor SIRPβ, another member of the SIRP family that mediates activation signaling in macrophages, might also contribute to the activation of TAMs, resulting in increased production of TNF (47). Furthermore, we detected upregulation of genes related to proinflammatory macrophages as well as oxidative phosphorylation in TAMs of mice treated with both SE12C3 and rituximab. Although oxidative phosphorylation in macrophages has been thought to be related to anti-inflammatory responses (48), it was recently implicated in proliferative and inflammatory responses (49). Our preclinical models thus suggest that SE12C3 promoted the reprogramming of TAMs from an anti-inflammatory to a proinflammatory phenotype *in vivo*.

Anti-hCD47 antibodies have also been investigated in clinical trials across various types of tumors including B cell lymphomas as therapeutics targeting the CD47-SIRPα axis (12). We indeed demonstrated that the anti-hCD47 antibody B6H12 in combination with rituximab markedly inhibited tumor growth in the HIS-MITRG mouse model (**Supplementary Figure S7A**). Thus, our model can also be useful for the preclinical evaluation of therapeutics targeting human CD47 *in vivo*. By contrast, different from SE12C3, we observed that B6H12 alone induced phagocytosis of Raji cells by both human and mouse macrophages, in addition to enhanced ADCP activity of rituximab *in vitro* (**Supplementary Figures S7B–D**). Thus, the anti-tumor effect of B6H12 is not only human macrophage–specific, but mouse macrophages are also directly involved in such effect through the binding of anti-hCD47 antibodies with Fcγ receptor on mouse macrophages. Our preclinical tumor model is thus likely unsuitable to evaluate the human macrophage-dependent effect of anti-hCD47 antibodies, and we therefore mainly focused on the anti-tumor effect of anti-hSIRPα antibodies in the present study.

PDX models are thought to accurately represent the biology and therapeutic responses of various tumors, including B cell lymphoma (50). PDX models of DLBCL were thus found to recapitulate the molecular features of primary tumors (27). We have now shown that HIS-MITRG mice supported the growth of DLBCL PDX tumor tissue to a greater extent than did MITRG mice, similar to previous findings for HIS-MISTRG mouse recipients of primary human DLBCL tumor tissue (36), indicating that human immune cells promote the engraftment of patient-derived tumor specimens *in vivo*. Moreover, we found that combination treatment with SE12C3 and rituximab exerted a greater antitumor effect and increased the number of human tumor-infiltrated macrophages to a greater extent compared with rituximab monotherapy in our PDX model. These results thus provide further support for the therapeutic potential of antibodies to hSIRPα, similar to that of anti-hCD47, for the treatment of DLBCL in combination with an antibody to hCD20 (51).

In conclusion, our preclinical tumor models have allowed us to evaluate the antitumor effect of immunotherapy targeting human macrophages, with a focus on the promotion of ADCP activity and reprogramming of TAMs (**Supplementary Figure S8**). Together with clinical trials, these models may therefore contribute to the development of therapeutics for DLBCL (52). However, these models still have several limitations with regard to their being considered as human syngeneic tumor models, particularly with regard to treatment with anti-hSIRPα. First, although we focused on MITRG mice rather than MISTRG mice, in which hSIRPα is expressed on endogenous mouse macrophages (22), we cannot exclude the possibility of indirect activation of mouse macrophages by SE12C3 treatment. Indeed, rituximab alone promoted phagocytosis of Raji cells by mouse macrophages *in vitro*, and rituximab treatment inhibited CDX tumor growth in nonhumanized MITRG mice. Activated human macrophages induced by treatment with rituximab and SE12C3 may therefore also promote the activation of endogenous mouse macrophages in our models. Second, it remains unclear how or the extent to which tumor-infiltrating T and natural killer (NK) cells contribute to antitumor responses—in particular, after treatment with anti-hSIRPα (53). Given the insufficient T cell selection and differentiation in the thymus of HIS mice (54), antigen-specific T cell response is thought to be minimal in our model. On the other hand, we previously reported that HIS-MISTRG mice promote human NK cell differentiation through the production of hIL-15 by human macrophages (22), and we indeed observed the infiltration of NK cells in the tumor of HIS-MITRG mice. Thus, human NK cells are likely implicated in the induction of rituximab-mediated cellular cytotoxicity in our model. Third, the extent of infiltration of human dendritic cells (DCs) into tumors was found to be low for HIS-MITRG mice, in part because of the consumption of Flt3L by endogenous mouse DCs (55). Our models might therefore not recapitulate the antitumor effect of SE12C3 dependent on DCs—in particular, that mediated by the cGAS-STING pathway, which is activated by anti-CD47 in a syngeneic mouse tumor model (56). Further modification of our models will thus be required to establish them as human syngeneic tumor models for *in vivo* evaluation of therapeutics that target human macrophages.

## Data availability statement

The bulk RNA-seq data generated in this study are publicly available in the Genomic Expression Archive (GEA) database (accession no. GSE239693). All other data generated in this study are available upon request from the corresponding author.

## Ethics statement

The studies involving humans were approved by Ethics board of Kobe University Graduate School of Medicine. The studies were conducted in accordance with the local legislation and institutional requirements. The participants provided their written informed consent to participate in this study. The animal study was approved by Kobe University Animal Experimentation Regulatory Committee. The study was conducted in accordance with the local legislation and institutional requirements.

## Author contributions

YS: Conceptualization, Data curation, Formal Analysis, Funding acquisition, Investigation, Writing – original draft, Writing – review & editing. RI: Formal Analysis, Funding acquisition, Writing – original draft, Writing – review & editing. TA: Formal Analysis, Resources, Writing – original draft. AR: Formal Analysis, Writing – original draft. DH: Formal Analysis, Writing – original draft. SK: Formal Analysis, Writing – original draft. SO: Resources, Writing – original draft. TT: Formal Analysis, Writing – original draft. OO: Formal Analysis, Writing – original draft. TK: Validation, Writing – original draft. YF: Resources, Writing – original draft. YK: Resources, Writing – original draft. YM: Validation, Writing – original draft. KY: Resources, Writing – review & editing. HMa: Resources, Writing – review & editing. HMi: Resources, Writing – review & editing. HY: Resources, Writing – review & editing. MM: Methodology, Writing – review & editing. TM: Funding acquisition, Supervision, Writing – review & editing.
