# Supplementary material for: Preclinical evaluation of the efficacy of an antibody to human SIRPα for cancer immunotherapy in humanized mouse models
Source: Front Immunol. 2023 Dec 14;14:1294814. doi: 10.3389/fimmu.2023.1294814 (PMC10757636; doi:10.3389/fimmu.2023.1294814)
Supplement: Supplementary file 1 [file DataSheet_1.pdf]

Supplementary Figures and Tables

Figure S1: Human immune cells promote tumor growth in HIS-MITRG mice.

Figure S2: The hSIRPα antibody SE12C3 promotes rituximab-induced inhibition of B cell lymphoma growth in HIS-MITRG mice.

Figure S3: Treatment with rituximab either alone or together with SE12C3 reduces the number of human B cells in tumor-bearing HIS-MITRG mice.

Figure S4: Importance of human macrophages for the inhibition of tumor growth by SE12C3-rituximab in HIS-MITRG mice.

FigureS5: SE12C3 and rituximab alter the phenotype of human tumor-infiltrating macrophages in vivo.

Figure S6: HIS-MITRG mice support primary DLBCL engraftment and SE12C3 enhances the antitumor effect of rituximab in a PDX model.

Figure S7: The hCD47 antibody B6H12 and rituximab inhibit B cell lymphoma growth *in vivo* in HIS-MITRG mice and promoted tumor cell phagocytosis *in vitro*.

Figure S8: In vivo tumor model for preclinical evaluation of the anti-tumor effect of antibodies to human SIRPα targeting human macrophages using HIS-MITRG mice.

Table S1. List of antibodies used for flow cytometric and histological analysis.

Table S2. Primer sequences for qPCR analysis.

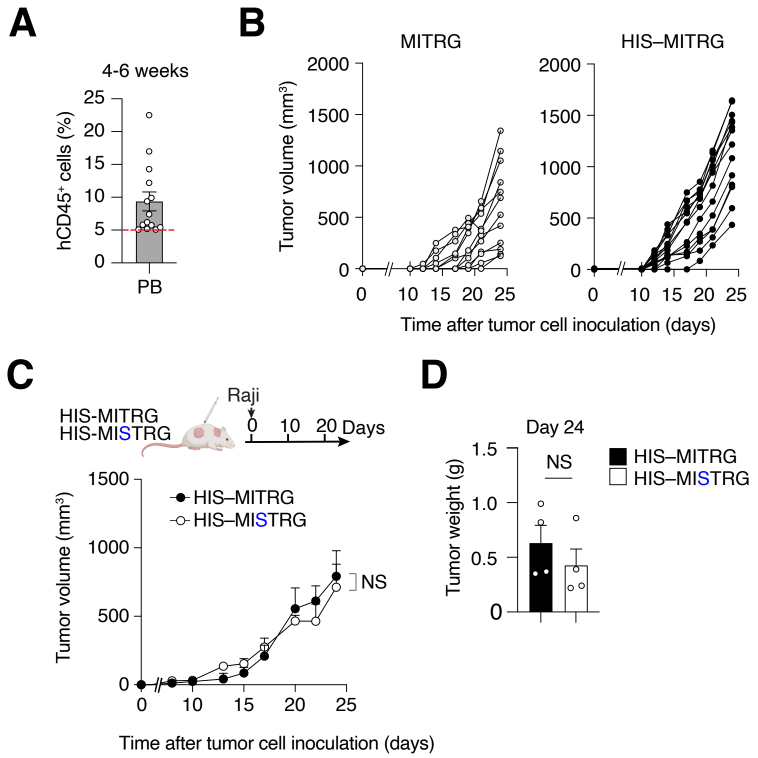

Supplementary Figure S1. Human immune cells promote tumor growth in HIS-MITRG mice. A, Percentage of $hCD45^+$ cells among total (human and mouse) $CD45^+$ cells in PB of HIS-MITRG mice (n = 14) at 4 to 6 weeks after $hCD34^+$ cell transplantation. The red dashed line indicates the cutoff value of 5% for successful engraftment. **B,** Tumor volume for individual HIS-MITRG (n = 14) or MITRG (n = 12) mice at the indicated times after Raji cell injection as in Figure 1B. **C,** Tumor volume in HIS-MITRG or HIS-MISTRG mice at the indicated times after Raji cell injection. **D,** Tumor weight for HIS-MITRG or HIS-MISTRG mice at 24 days after Raji cell injection. Data in **C** and **D** are means + SEM for four mice per group examined in two experiments. NS, not significant [two-way repeated-measures ANOVA followed by Šídák's test (**C**), or Student's *t* test (**D**)].

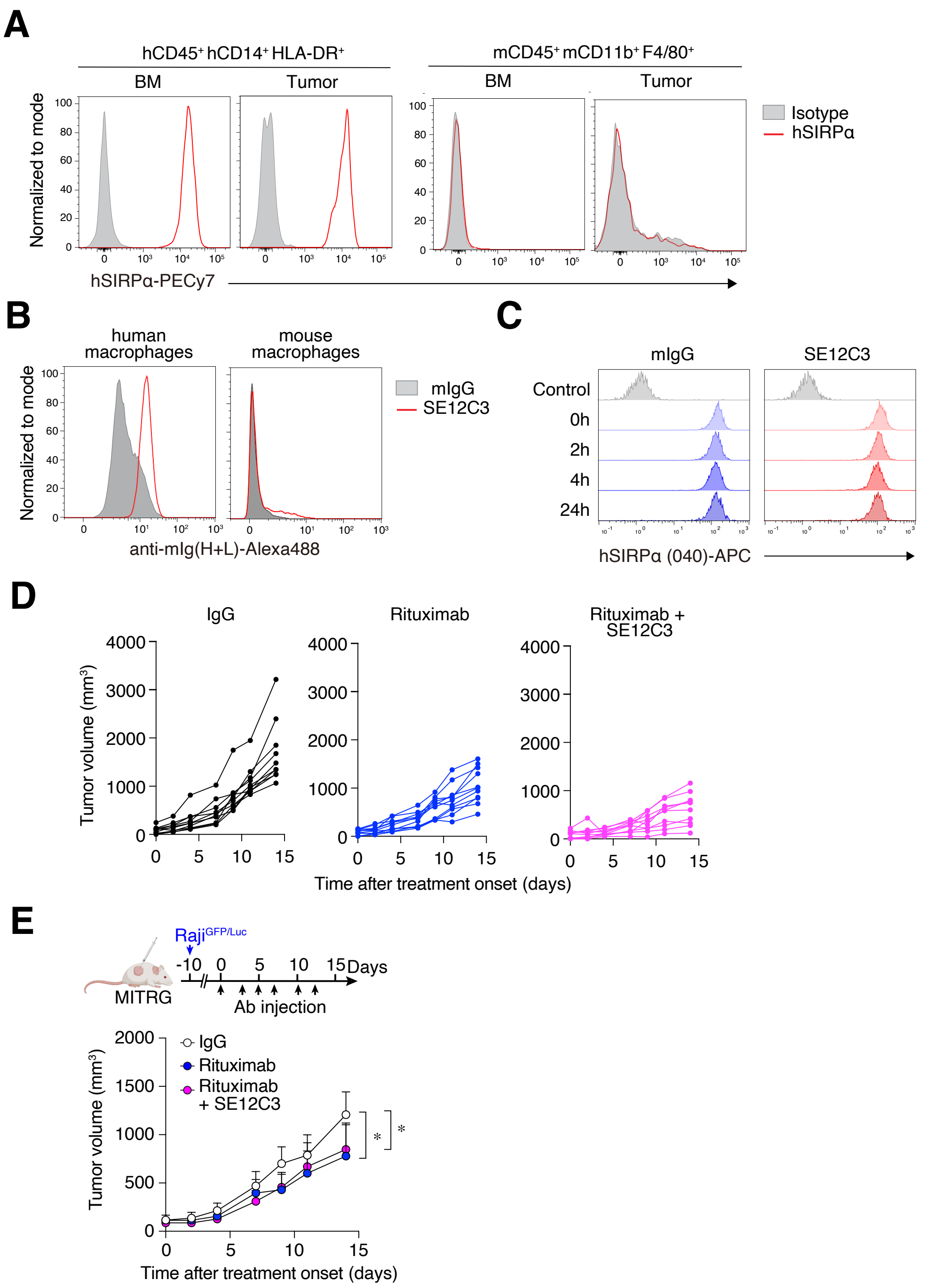

Supplementary Figure S2. The hSIRPα antibody SE12C3 promotes rituximab-induced inhibition of B cell lymphoma growth in HIS-MITRG mice. A, Expression of human SIRPα on human macrophages (hCD45^{+}hCD14^{+}HLA-DR^{+}) and mouse macrophages (mCD45^{+}mCD11b^{+}F4/80^{+}) in the BM or tumor of HIS-MITRG mice bearing Raji tumors at 24 days after tumor injection. Data are representative of five mice per group. **B**, Biding of control mIgG or SE12C3 on CB–derived human macrophages (left) or MITRG BM–derived mouse macrophages (right) measured by the

staining with Alexa488 anti-mIg(H+L) secondary antibodies. Human macrophages were preincubated with hIgG and mouse macrophages were with anti-mCD16/32 to minimize the engagement of antibodies with Fc receptors. Data are representative of triplicate samples. **C**, CB–derived human macrophages were incubated with control mIgG (left) or SE12C3 (right). Cells were harvested at indicated time points and stained with biotin anti-hSIRPα antibody (clone 040), followed by the staining with streptavidin APC. Data are representative of triplicate samples. **D,** Time course of tumor volume for individual HIS-MITRG mice bearing $Raji^{GFP/Luc}$ tumors and treated with control IgG (n = 10), rituximab (n = 11), or rituximab plus SE12C3 (n = 10) as in Figure 2B. **E,** Time course of tumor volume for MITRG mice bearing $Raji^{GFP/Luc}$ tumors and treated with control IgG (n = 4), rituximab (n = 5), or rituximab plus SE12C3 (n = 5). Data are means + SEM for mice examined in two experiments. *$P < 0.05$ (two-way repeated-measures ANOVA followed by Šídák's test).

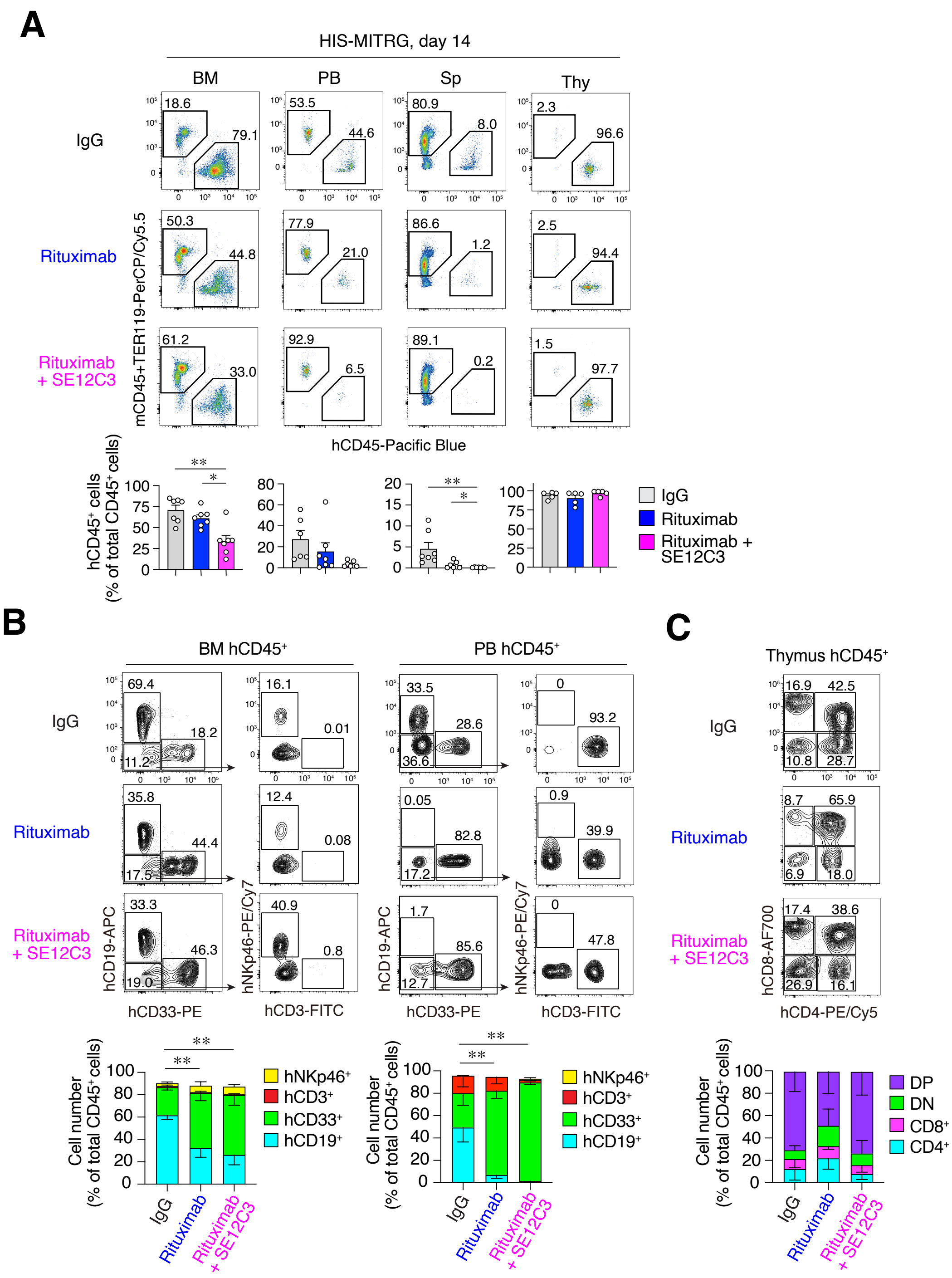

Supplementary Figure S3. Treatment with rituximab either alone or together with SE12C3 reduces the number of human B cells in tumor-bearing HIS-MITRG mice. A, Representative FACS plots for hCD45⁺ and mCD45⁺ (and TER119⁺ for spleen) cells in BM, PB, spleen (Sp), or thymus (Thy) of HIS-MITRG mice bearing Raji$^{GFP/Luc}$ tumors at 14 days after the onset of antibody treatment as in Figure 2A. The percentages of hCD45⁺ cells among total (h + m) CD45⁺ (and TER119⁺ for spleen) cells are also

shown below the FACS plots. **B,** Representative FACS plots for human immune cells in BM and PB for mice as in **A**. The percentages of $hCD19^+$, $hCD33^+$, $hCD3^+$, or $hNKp46^+$ cells among $hCD45^+$ cells are also shown below the FACS plots. **C,** Representative FACS plots for $CD4^-CD8^-$ (DN), $CD4^+CD8^+$ (DP), and $CD4^+$ or $CD8^+$ single-positive (SP) thymocyte subsets for mice as in **A**. The percentages of DN, DP, $CD4^+$ SP, and $CD8^+$ SP subsets among $hCD45^+$ cells are also shown below the FACS plots. All data in bar graphs are means ± SEM for $n = 7$ mice per group examined in four experiments. $*P < 0.05$, $**P < 0.01$ [one-way ANOVA followed by Tukey's test (**A**), or two-way ANOVA followed by Tukey's test (**B**)].

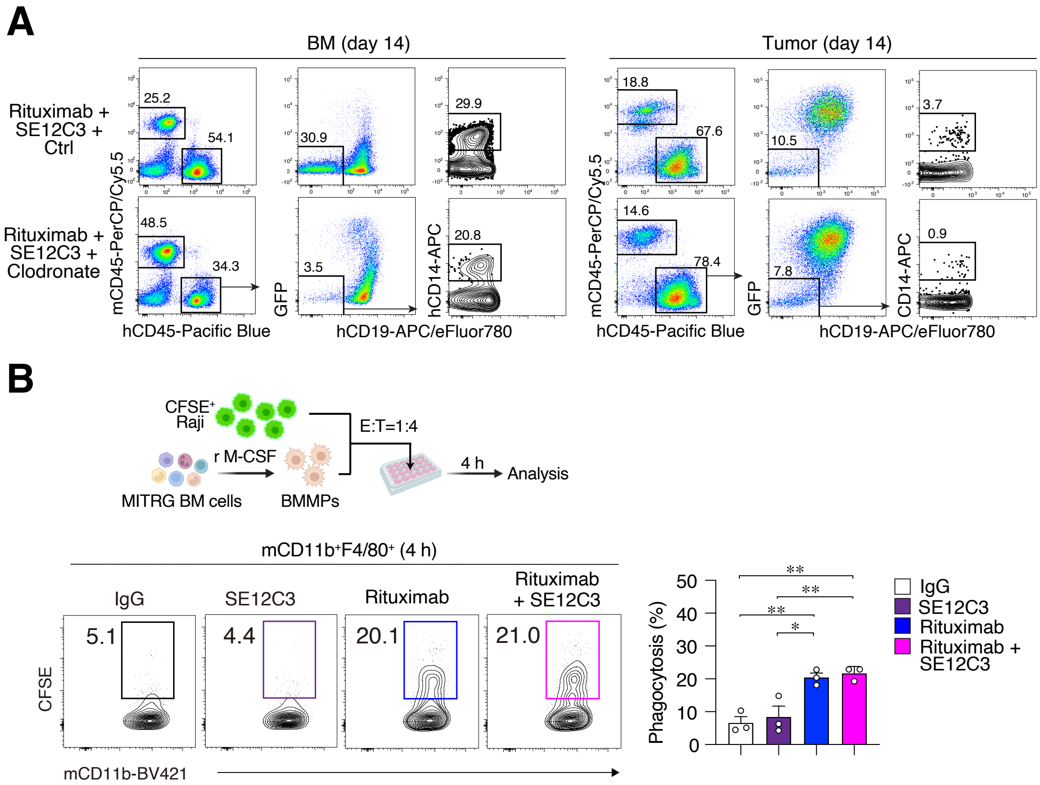

Supplementary Figure S4. Importance of human macrophages for the inhibition of tumor growth by SE12C3-rituximab in HIS-MITRG mice. A, Representative FACS plots for hCD45⁺ cells (left), GFP⁻hCD19⁻ cells (middle), and hCD14⁺ cells (right) in BM or PB of HIS-MITRG mice bearing Raji$^{GFP/Luc}$ tumors and injected with PBS liposomes (Ctrl, *n* = 7) or clodronate liposomes (*n* = 7) as shown in Figure 4B. **B,** CFSE-labeled Raji cells were incubated for 4 h with BM-derived macrophages (BMMPs) from MITRG mice and in the presence of the indicated antibodies. The cells were then analyzed by flow cytometry as indicated. The proportion of CFSE⁺mCD11b⁺ cells among total mCD11b⁺ cells was determined as percentage phagocytosis. Representative results and averaged data (means + SEM) for three or four independent experiments are shown.

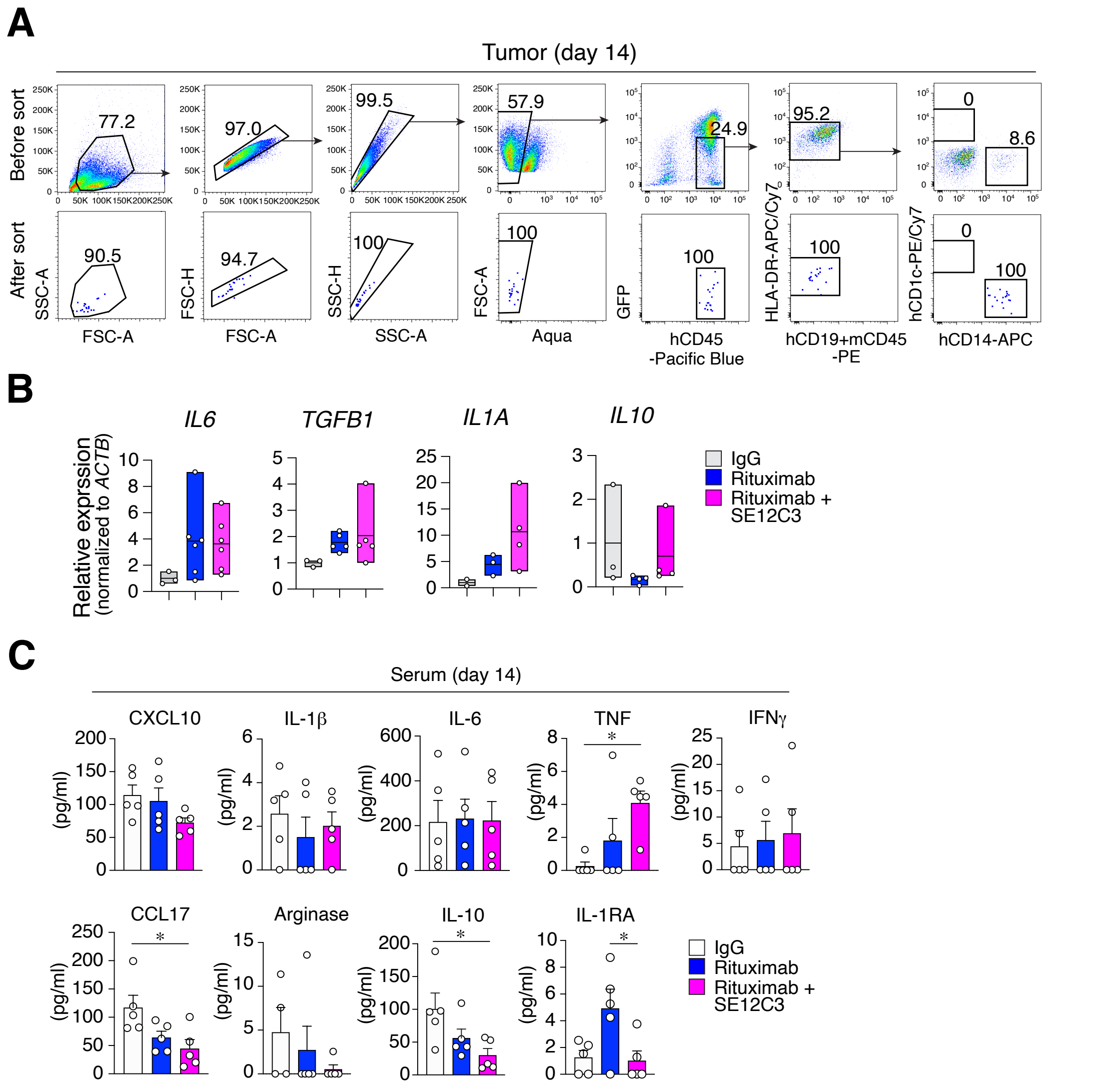

Supplementary Figure S5. SE12C3 and rituximab alter the phenotype of human tumor-infiltrating macrophages *in vivo*. A, Gating strategy for sorting of human tumor-infiltrating macrophages from mice treated as in Figure 5A. The sorted cells were then subjected to the same procedure in order to confirm their purity. FSC, forward scatter; SSC, side scatter. **B,** RT-qPCR analysis of gene expression in the sorted cells from mice treated with control IgG (n = 2 or 4), rituximab (n = 3 to 6), or rituximab plus SE12C3 (n = 4 to 7). Data are presented as box plots for mice examined in four independent experiments. **C,** Concentrations of human cytokines and chemokines in serum of Raji$^{GFP/Luc}$ tumor–bearing HIS-MITRG mice at 14 days after the onset of treatment with control IgG (n = 5), rituximab (n = 5), or rituximab plus SE12C3 (n = 5). Data are means + SEM from three experiments. *$P < 0.05$ (one-way ANOVA followed by Tukey's test).

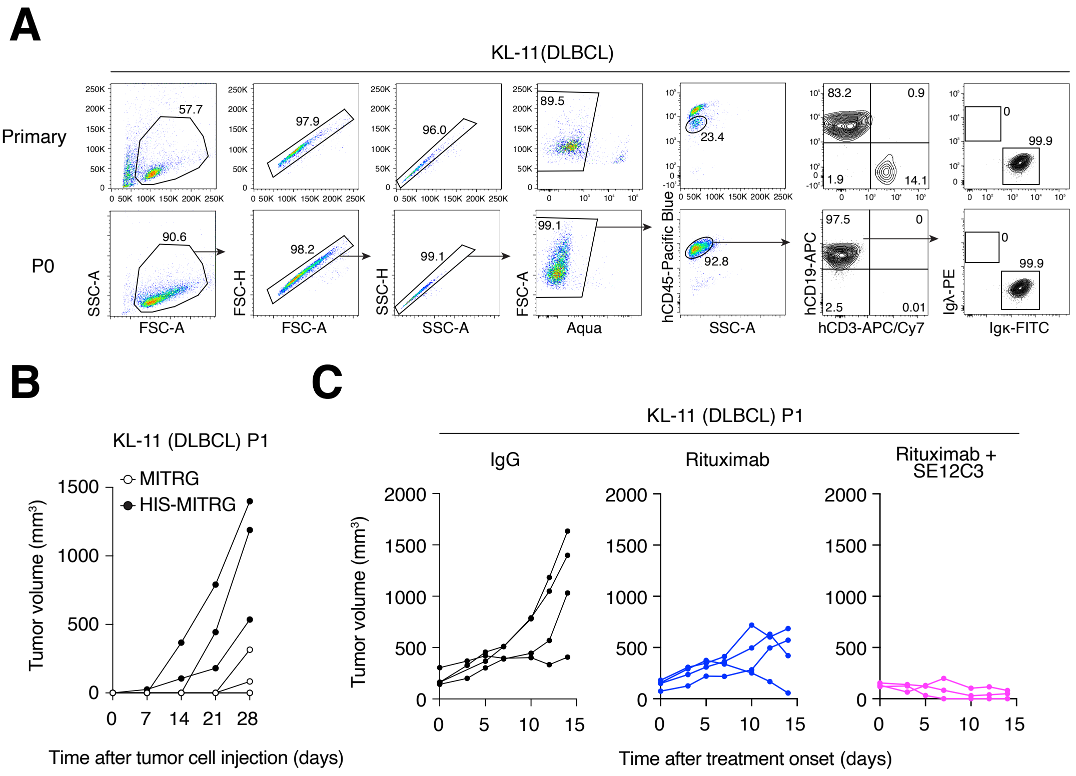

Supplementary Figure S6. HIS-MITRG mice support primary DLBCL engraftment and SE12C3 enhances the antitumor effect of rituximab in PDX model. A, Flow cytometric analysis of the expression of Igλ and Igκ in hCD45^{lo}hCD19^{+} viable cells isolated from primary or P0 DLBCL tumor tissue as in Figure 6B. Data are representative of two independent experiments. **B,** Tumor volume for individual HIS-MITRG (*n* = 3) or MITRG (*n* = 4) mice in Figure 6C. **C,** Tumor volume for individual HIS-MITRG mice treated with control IgG (*n* = 4), rituximab (*n* = 4), or rituximab plus SE12C3 (*n* = 3) in Figure 6D.

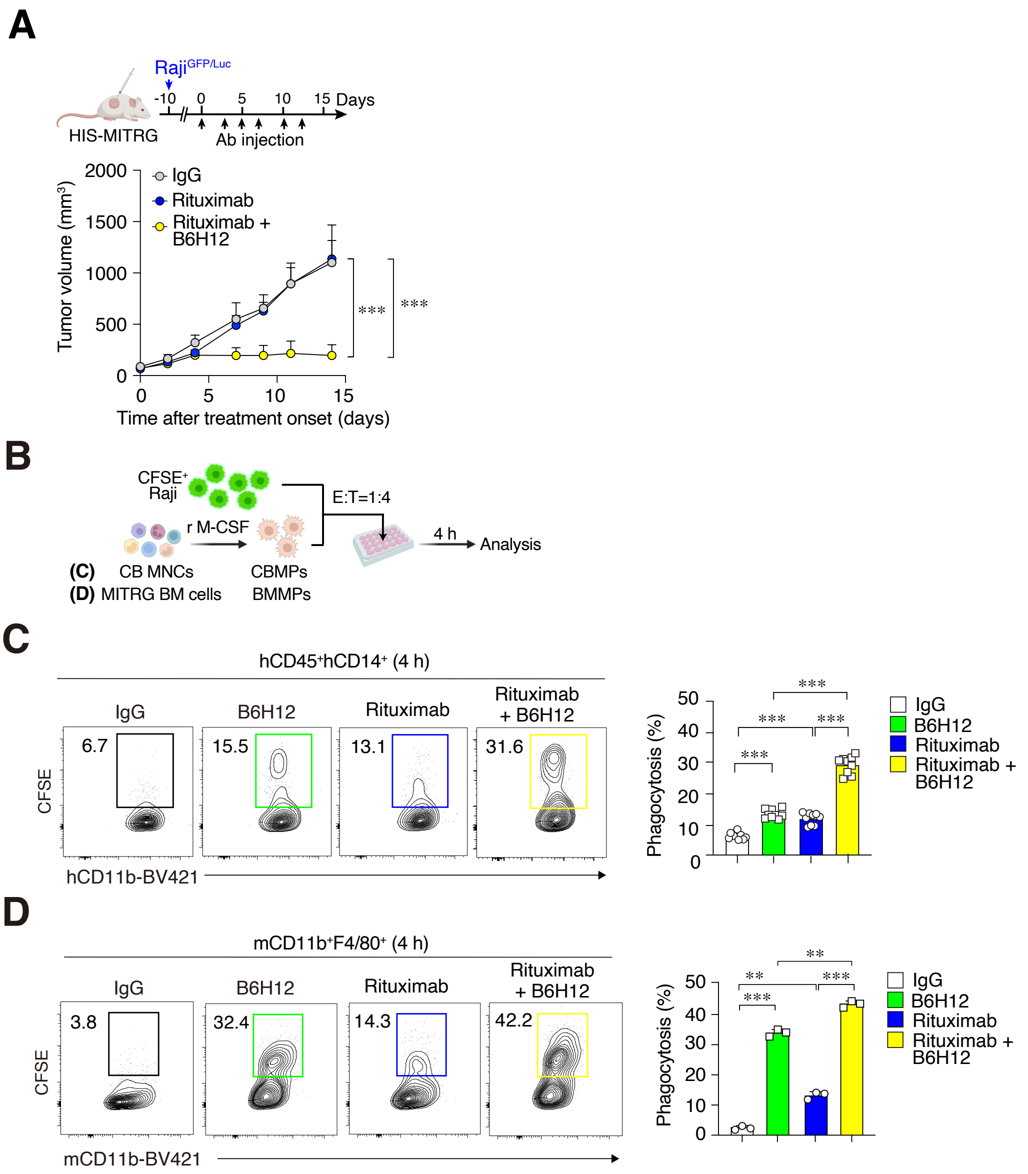

Supplementary Figure S7. The hCD47 antibody B6H12 and rituximab inhibit B cell lymphoma growth *in vivo* in HIS-MITRG mice and promoted tumor cell phagocytosis *in vitro*. A, Time course of tumor volume for MITRG mice bearing Raji$^{GFP/Luc}$ tumors and treated with control IgG (n = 4), rituximab (n = 5), or rituximab plus anti-hCD47 antibody B6H12 (n = 5). Data are means + SEM for mice examined in two experiments. **B,** Experimental scheme of *in vitro* phagocytosis assay. **C,** CFSE-labeled Raji cells were incubated for 4 h with human CBMPs in the presence of the indicated antibodies. the proportion of CFSE^{+}CD11b^{+} cells among total CD11b^{+} cells was determined by flow cytometry as percentage phagocytosis as indicated. Representative results and averaged data (means + SEM) from three independent experiments are shown. **D,** CFSE-labeled Raji cells were incubated for 4 h with

BMMPs from MITRG mice and in the presence of the indicated antibodies. The cells were then analyzed by flow cytometry as indicated. The proportion of CFSE^{+}mCD11b^{+} cells among total mCD11b^{+} cells was determined as percentage phagocytosis. Representative results and averaged data (means + SEM) for two independent experiments are shown. $^{**}P < 0.01$, $^{***}P < 0.001$ (two-way repeated-measures ANOVA followed by Šídák's test **(A)** or one-way ANOVA followed by Tukey's test **(C, D)**).

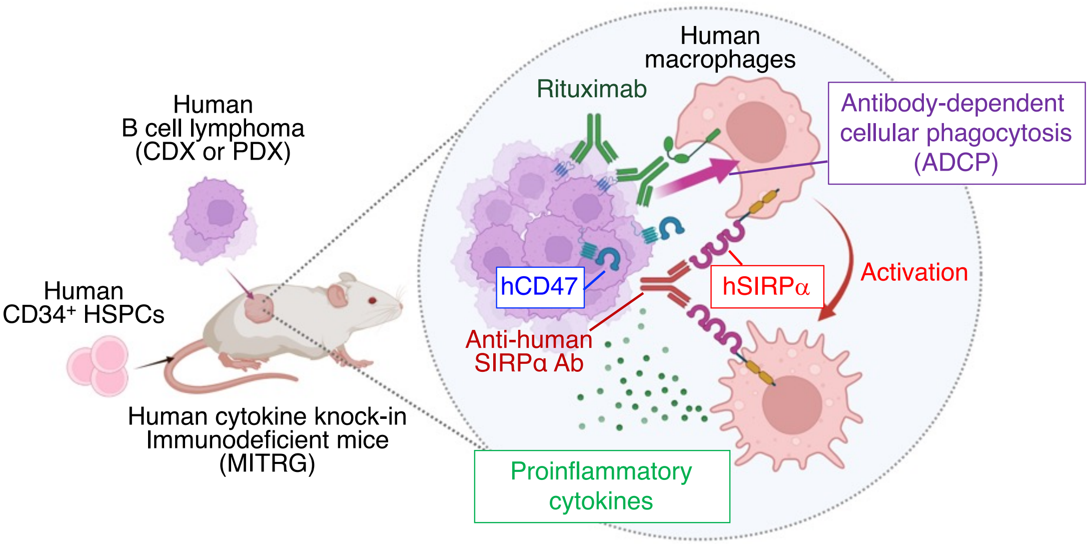

Supplementary Figure S8. In vivo tumor model for preclinical evaluation of the anti-tumor effect of antibodies to human SIRPα targeting human macrophages using HIS-MITRG mice. HIS-MITRG mice, which are generated by the transplantation of $CD34^{+}$ hematopoietic stem and progenitor cells (HSPCs) into MITRG mice, are subcutaneously inoculated with human B cell lymphoma and subsequently treated with rituximab and anti-human SIRPα antibodies. The combination therapy enhanced antibody-dependent cellular phagocytosis (ADCP) of tumor cells by human macrophages as well as the activation of human macrophages to secrete proinflammatory cytokines, both of which resulted in the eradication of tumor cells by human macrophages. CDX, cell line–derived xenograft; PDX, patient–derived xenograft.

Supplementary Table S1. List of antibodies used for flow cytometric and histological analysis

Antibody	Clone	Source
Mouse anti-human CD45 Pacific Blue	HI30	BioLegend
Rat anti-mouse CD45 PerCP-Cy5.5	30-F11	BioLegend
Rat anti-mouse CD45 BV711	30-F11	BD Biosciences
Rat anti-mouse CD45 Biotin	30-F11	BioLegend
Mouse anti-human CD19 APC	HIB19	BioLegend
Mouse anti-human CD19 APC-eFluor780	HIB19	Thermo Fisher (eBioscience)
Mouse anti-human CD19 PerCP-Cy5.5	HIB19	Cytek (TONBO Biosciences)
Mouse anti-human CD19 Biotin	HIB19	BioLegend
Mouse anti-human CD33 PE	WM53	BioLegend
Mouse anti-human CD3 FITC	HIT3a	BioLegend
Mouse anti-human CD3 APC-Cy7	HIT3a	BioLegend
Mouse anti-human CD3 PerCP-Cy5.5	OKT3	Cytek (TONBO Biosciences)
Mouse anti-human CD3 BV785	OKT3	BioLegend
Mouse anti-human CD4 PE-Cy5	OKT4	BioLegend
Mouse anti-human CD8 AF700	HIT8a	BioLegend
Mouse anti-human NKp46 PE-Cy7	9E2	BioLegend
Mouse anti-human CD14 APC	M5E2	BioLegend
Mouse anti-human CD11b PE-CF594	ICRF44	BioLegend
Mouse anti-human HLA-DR APC-Cy7	L243	BioLegend
Mouse anti-human HLA-DR BV605	L243	BioLegend
Mouse anti-human CD1c PE-Cy7	L161	BioLegend
Mouse anti-human SIRPα/β	SE5A5	BioLegend
Rabbit anti-human ϰ light chains FITC / Rabbit anti-human λ light chains RPE	Polyclonal	DAKO
Rat anti-mouse/human CD11b BV421	M1/70	BioLegend
Rat anti-mouse F4/80 PE	BM8	BioLegend
Rat anti-mouse TER119 PerCP-Cy5.5	TER119	BioLegend
Streptoavidin PE	–	Cytek (TONBO Biosciences)
Streptoavidin APC	–	BioLegend
Goat anti-mouse IgG (H+L) AF488	Polyclonal	Jackson ImmunoResearch
Human TruStain FcX™ (mouse anti-human CD16, CD32, and CD64)	3G8, FUN-2, 10.1	BioLegend

Rat anti-mouse CD16/32	96	BioLegend
Rabbit anti-human CD68, purified	KP1	Cell Signal Technology
Mouse anti-human CD163, purified	10D6	Leica

AF, AlexaFluor; APC, allophycocyanin; BV, Brilliant Violet; CF, carboxyfluorescein; Cy, cyanin; FITC, fluorescein isothiocyanate; PE, phycoerythrin; PerCP, peridinin-chlorophyll protein complex.

Supplementary Table S2. Primer sequences for qPCR analysis

Primer	Forward (5′→3′)	Reverse (5′→3′)
ACTB	ACA ATG AGC TGC GTG TGG CT	TCT CCT TAA TGT CAC GCA CGA
IL1B	TCG AGG CAC AAG GCA CAA C	TGT TTA GGG CCA TCA GCT TCA
TNF	GAG GCC AAG CCC TGG TAT G	CGG GCC GAT TGA TCT CAG C
PTGS2	TCT TTG CCC AGC ACT TCA CG	AAG GCG CAG TTT ACG CTG TC
IL10	CAA GAC CCA GAC ATC AAG GCG	GGC ATT CTT CAC CTG CTC CAC
TGFB1	CGA CTC GCC AGA GTG GTT AT	CGG TAG TGA ACC CGT TGA TGT
IL6	GGA GAC TTG CCT GGT GAA A	CTG GCT TGT TCC TCA CTA CTC
IL1A	ATC AGT ACC TCA CGG CTG CT	TGG GTA TCT CAG GCA TCT CC
CXCL5	GAG AGC TGC GTT GCG TTT G	TTT CCT TGT TTC CAC CGT CCA
CD274	GGC ATC CAA GAT ACA AAC TCA A	CAG AAG TTC CAA TGC TGG ATT A
